# Dynamic nanopore long-read sequencing analysis of HIV-1 splicing events during the early steps of infection

**DOI:** 10.1186/s12977-020-00533-1

**Published:** 2020-08-17

**Authors:** Nam Nguyen Quang, Sophie Goudey, Emmanuel Ségéral, Ammara Mohammad, Sophie Lemoine, Corinne Blugeon, Margaux Versapuech, Jean-Christophe Paillart, Clarisse Berlioz-Torrent, Stéphane Emiliani, Sarah Gallois-Montbrun

**Affiliations:** 1 Institut Cochin, INSERM, CNRS, Université de Paris, 75014 Paris, France; 2 Genomic Facility, Institut de Biologie de l’ENS (IBENS), Département de biologie, École normale supérieure, CNRS, INSERM, Université PSL, 75005 Paris, France; 3 CNRS, Architecture et Réactivité de l’ARN, UPR 9002, IBMC, Université de Strasbourg, Strasbourg, France

**Keywords:** HIV RNA, Alternative splicing, Viral transcriptome, ONT long-read sequencing

## Abstract

**Background:**

Alternative splicing is a key step in Human Immunodeficiency Virus type 1 (HIV-1) replication that is tightly regulated both temporally and spatially. More than 50 different transcripts can be generated from a single HIV-1 unspliced pre-messenger RNA (pre-mRNA) and a balanced proportion of unspliced and spliced transcripts is critical for the production of infectious virions. Understanding the mechanisms involved in the regulation of viral RNA is therefore of potential therapeutic interest. However, monitoring the regulation of alternative splicing events at a transcriptome-wide level during cell infection is challenging. Here we used the long-read cDNA sequencing developed by Oxford Nanopore Technologies (ONT) to explore in a quantitative manner the complexity of the HIV-1 transcriptome regulation in infected primary CD4+ T cells.

**Results:**

ONT reads mapping to the viral genome proved sufficiently long to span all possible splice junctions, even distant ones, and to be assigned to a total of 150 exon combinations. Fifty-three viral RNA isoforms, including 14 new ones were further considered for quantification. Relative levels of viral RNAs determined by ONT sequencing showed a high degree of reproducibility, compared favourably to those produced in previous reports and highly correlated with quantitative PCR (qPCR) data. To get further insights into alternative splicing regulation, we then compiled quantifications of splice site (SS) usage and transcript levels to build “splice trees”, a quantitative representation of the cascade of events leading to the different viral isoforms. This approach allowed visualizing the complete rewiring of SS usages upon perturbation of SS D2 and its impact on viral isoform levels. Furthermore, we produced the first dynamic picture of the cascade of events occurring between 12 and 24 h of viral infection. In particular, our data highlighted the importance of non-coding exons in viral RNA transcriptome regulation.

**Conclusion:**

ONT sequencing is a convenient and reliable strategy that enabled us to grasp the dynamic of the early splicing events modulating the viral RNA landscape in HIV-1 infected cells.

## Background

By producing a variety of different mRNA isoforms, alternative splicing is a means for the majority of cellular genes to expand and regulate expression of novel protein isoforms [1]. Splicing is a two-step catalytic process orchestrated by the spliceosome, a dynamic ribonucleoprotein complex comprised of five small nuclear RNAs (snRNAs) (U1, U2, U4, U5 and U6) and a large number of protein factors [2]. Assembly of the spliceosome occurs on pre-mRNA via recognition of specific RNA elements including the splice donor (SD) site at the 5′-end of the intron, the splice acceptor (SA) site at the 3′-end and the branch-site, and leads to excision of the intron [3].

During its replication, HIV-1 utilises this cellular process to generate more than 50 different viral transcripts from a single pre-mRNA [4, 5]. This highly regulated process impacts both the production of unspliced (US) genomic RNA and the mRNAs coding for viral proteins [6–8]. In most HIV-1 strains, alternative splicing relies on four major SD (D1 to D4) and eight SA (A1, A2, A3, A4a, A4b, A4c, A5 and A7) sites and competition in the usage of these splice sites generates the full range of mRNAs [8, 9]. HIV-1 transcripts are typically classified according to their size: the 9-kilobase (kb) US RNA that is either translated into Gag/Gag-Pol polyproteins, or directly packaged into viral particles as genomic RNA; the 4-kb incompletely spliced (IS) RNAs that usually result from splicing events between the major SD site D1 and one of the SA sites: A1 for Vif, A2 for Vpr and A4 and A5 for Env/Vpu; and the 2-kb multiply spliced (MS) RNAs produced by an additional splicing event between D4 and A7, encoding Tat, Rev and Nef proteins. More complexity in SS usage is added by two suboptimal SD sites D2 and D3 that allow the production of two small non-coding exons (NCE) 2 and 3, which can be included in both the 2-kb and 4-kb transcripts. Cryptic SS and unusual sites have also been described in several HIV-1 isolates/subtypes, expending further the repertoire to more than a hundred possible isoforms produced during infection [4, 5, 10–13].

The decision as to which exon is included or removed depends on the intrinsic strength of SD and SA sites, viral RNA structures, and the influence of RNA regulatory sequences in the nearby exon or intron that allow the recruitment of splice activator or splice inhibitor factors [7, 9, 12, 14–25].

Production of spliced isoforms is tightly regulated during viral replication: MS transcripts are rapidly produced in the early phases of infection. As this later progresses, there is a shift towards production of IS and US transcripts [6, 26, 27]. This shift is dependent on the threshold level of the Rev protein, which facilitates the export of US and IS RNAs from the nucleus by binding to the RRE (Rev-response element), a stem-loop structure located between D4 and A7 in the Env coding region of IS and US transcripts [28–30]. Imbalance in the tight spatiotemporal specificity of viral RNA production can have dramatic effects on HIV-1 viral expression: a diminution of splicing reduces the level of key regulatory proteins encoded by MS RNAs that are necessary for viral transcription and RNA nuclear export, while an excess of splicing reduces the levels of US and IS RNAs encoding structural, enzymatic and accessory proteins [4, 31–33]. Unravelling splicing at the molecular level is thus key to understanding HIV-1 replication.

However, with a wide range of RNA sizes and expression levels the HIV-1 transcriptome has proved challenging to study. Southern blot and semi-quantitative reverse transcriptase (RT)-PCR using specific primers for the 2-kb and 4-kb transcripts were first used to explore the panel of viral isoforms produced during infection and determine their relative abundance [4, 34, 35]. The arrival of next generation sequencing (NGS) that allows the analysis of millions of splicing events has considerably enriched our knowledge on splicing regulation [36]. In the last 10 years, high-throughput sequencing technologies have increased the repertoire of HIV-1 RNAs produced during infection from around 50 transcripts to more than a hundred [4, 5, 10–12]. Nevertheless, with an average read size of only 150 nucleotides NGS rarely queries more than one splice junction, restricting its ability to both resolve complex isoform patterns and apprehend the true extent of alternative splicing events. To overcome this limitation, an approach using single-molecule amplification in combination with the long-read PacBio sequencing was employed to analyze transcripts produced by T lymphocytes infected with the HIV-1_89.6_ isolate. This approach identified new SD and SA sites and a new class of 1-kb transcripts [5]. Recently, combined short-read sequencing with the use of primers specific for the 2-kb and 4-kb classes of HIV-1 RNAs provided new insights into the regulation of viral splicing elements [12, 17]. Nevertheless, both techniques suffer from the fact that transcript levels can only be compared within each of 2-, 4-, or 9-kb class giving incomplete views of the splicing events leading to the HIV-1 RNA repertoire.

To apprehend the full complexity of HIV-1 alternative splicing regulation in a quantitative manner, we applied the recently developed Nanopore sequencing from Oxford Nanopore Technologies on cDNA produced from HIV-1 expressing cells. ONT sequencing does not require fragmentation of RNA before sequencing, nor specific primers for library preparation or amplification. It has been shown to sequence short and ultra-long reads of hundreds of kilobases without bias for high or low GC contents [37]. ONT sequencing has been successfully applied to study alternative splicing of several human transcripts [38–40] and to explore viral transcriptomes [41–49]. We took advantage of this technology to produce HIV-1 reads long enough to cover all the possible splice junctions present on viral isoforms. Based on the composition of spliced junctions we could then resolve viral isoforms and produce a quantitative picture of the viral transcriptome produced in infected primary CD4+ T cells. By integrating both SS usage and isoform levels in splice tree representations, we then explored in detail the temporal plasticity of the viral transcriptome. Altogether we propose a powerful tool to follow the dynamic of HIV-1 alternative splicing and provide an unprecedented detailed view of the cascade of splicing events occurring during the early steps of HIV-1 infection.

## Results

### Sequencing viral transcripts from HIV-1 producing cells

To investigate the viral splicing pattern in HIV-1 producing cells, primary CD4+ T cells from three different donors were isolated and infected for 24 h with HIV NL4-3 viruses pseudotyped with the vesicular stomatitis virus glycoprotein (VSV-G). Total cellular RNA was extracted and was used to generate cDNA libraries for ONT sequencing. Between 575,784 and 741,005 reads were mapped to both the human and HIV NL4-3 genomes using *Minimap2* software (Additional file 1: Table S1). This alignment program works with both relatively short (> 100 nucleotides) and long reads, is compatible with high error rates generated by ONT sequencing and is faster than short-read aligners. By tolerating long insertions and deletions and allowing split-read alignments, it also proved to be adapted to splice alignment [50]. Depending on the T cell donor, between 4974 and 12,314 reads were mapped to HIV-1 genome, with stretches up to 7969 nucleotides long. This corresponds to a total of 4.87 × 10^6^ to 13.61 × 10^6^ aligned bases, i.e. covering more than 500 times the HIV NL4-3 genome. In agreement with other RNA sequencing studies, viral reads represented between 0.9 and 2% of total reads in infected T cells [27, 51–53] (Additional file 1: Table S1). As a comparison, the three cellular transcripts that are covered by the highest number of reads are B2M, TMSB10 and RPS29 with 0.62 to 1.31% of total reads, suggesting that HIV transcripts are highly expressed in infected CD4+ T cells (data not shown). Nanopore sequencing of non-infected, infected or transfected HeLa cells was also performed to further compare HIV-1 alternative splicing patterns across different models of HIV-1 producing cells (Additional file 2: Table S2).

### Detection of HIV-1 splice sites

**Fig. 1:**
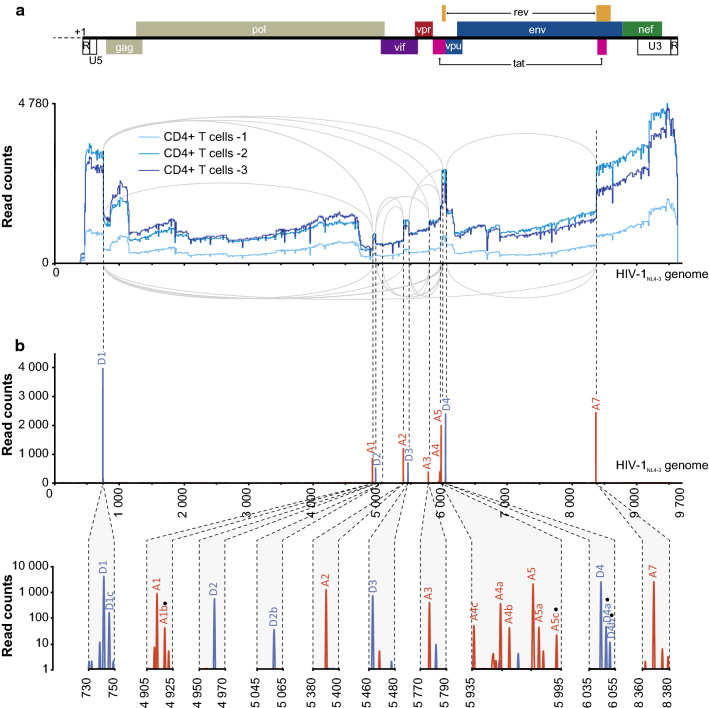
Annotation and usage of HIV-1 splice sites by ONT sequencing in infected T cells. **a** Sashimi plots for flanking and alternatively spliced exons determined by ONT sequencing in infected CD4+ T cells from 3 donors were visualized in *IGV*. Exon boundaries between putative SD and SA sites are symbolized in grey lines. Organization of HIV NL4-3 genome is indicated. **b** SD (in blue) and SA (in red) sites considered in the rest of the analysis are presented. Enlarged representations allow the observation of previously reported cryptic SS as well as putative new SS (indicated by a black dot). Values represent the sum of reads mapping to each SS across the three T cell samples

As illustrated by the Sashimi plot in Fig. 1a, the alignment of viral reads to the full-length NL4-3 sequence displayed a complex pattern in infected CD4+ T cells. Although the total number of reads varied from one donor to another, their coverage profiles were highly similar. Gaps in the sequences were considered as potential excised introns and events corresponding to splice junctions were indicated by grey lines. To identify SD and SA sites, start/end positions of putative exons from all reads were collected and counted (Fig. 1b and Additional file 3: Table S3). Despite a mean mismatch (indel + substitution) rate estimated around 11% in CD4+ T cells samples (Additional file 1: Table S1), the four major SD sites and the eight consensus SA sites were identified without ambiguity at their exact known location.

In agreement with previous studies, D1 and D4 were the most used SD sites, with 3970 and 2394 overlapping reads, respectively, whereas D2 and D3 were considered as suboptimal with 524 and 698 overlapping reads, respectively [5, 10, 12, 54]. Two previously reported cryptic SS, D1c and D2b, were also detected. Both sites are highly conserved amongst most HIV-1 strains, but were reported as weak SS, indicating that our assay allows the detection of relatively rare events [4, 5, 10, 12]. Amongst SA sites, A7 and A5 were the most represented, followed by A2, A1, A4a and A3, A4b and A4c (Fig. 1b). A5a, a cryptic SA site [5] was almost as represented as A4c in our assay.

In contrast, usage of cryptic SA sites A6, A7a, A7b and D5 identified in the HXB2 strain was barely detectable, confirming that these sites are rarely, if ever, used in the NL4-3 strain [4, 35]. Using the PacBio platform, Ocwieja et al. confirmed the presence of a set of SA sites located within the U3 region of HIV-1_89.6_ genomic RNA [5]. Although we identified junctions involving A8, A8b and A8f in NL4-3 RNAs, their occurrence was too low to be considered further.

In addition, four new potential SS were detected (Fig. 1b). Only SS comprising the canonical GU and AG dinucleotides, in exon combinations present in at least 2 independent biological replicates and at least 5 times across the replicates were considered as genuine [55, 56]. Two new putative SD sites were detected in the vicinity of D4 (D4a and D4b), as well as two new SA sites, A5c and A1b (Fig. 1b and Additional file 3: Table S3). Of note, although these potential SA and SD sites are infrequently used, they were also detected in infected and transfected HeLa cells (Additional file 3: Table S3). Moreover, consensus nucleotides, GT at D4a and AG at A1b, are very well conserved in HIV-1 subtype M (A + K recombinants) (conservation percentage = 99%) reinforcing the likelihood of these new sites.

### Viral transcript identification

Based on splicing events we went on to identify HIV-1 transcripts in our different models of HIV-1 producing cells. Considering that D1 is engaged in a splice junction in all spliced isoforms, reads mapping to intron 1 were assigned to US RNA. Amongst the reads harbouring splice junctions, reads too short to be assigned to a particular isoform without ambiguity were discarded. Thus, between 2629 and 6606 reads with a mean read length between 1058 and 1386 nucleotides were annotated in T cell samples (Additional file 1: Table S1).

In total, 150, 122 and 147 different exon combinations were detected in CD4+ T cells, infected HeLa cells and transfected HeLa cells, respectively (Additional file 4: Table S4 and Additional file 5: Figure S1). These comprised all HIV-1 transcripts originally described by Purcell et al. [4].

**Fig. 2:**
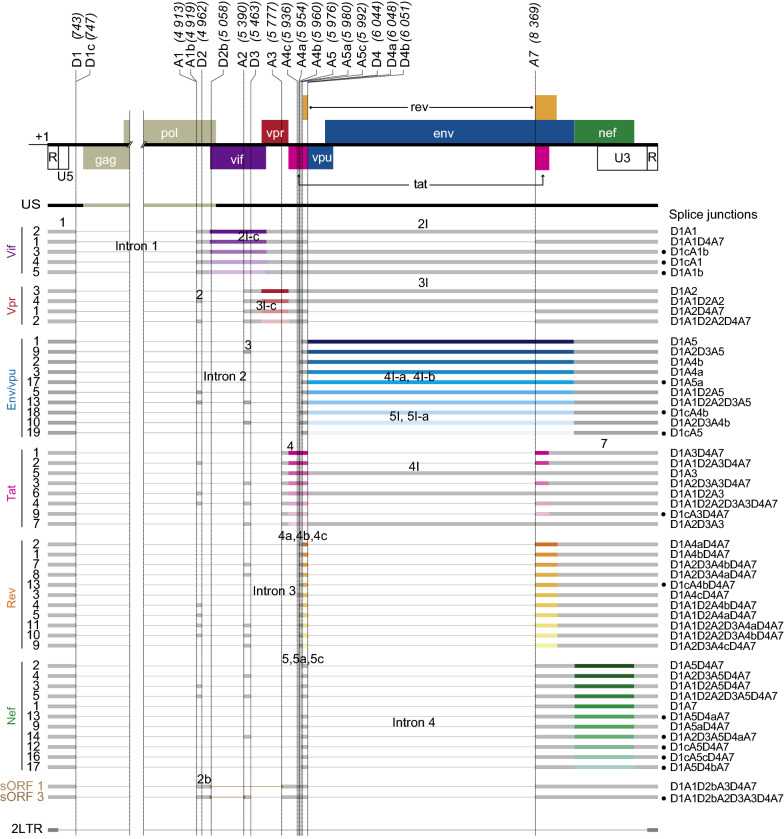
Schematic representation of HIV-1 RNA population detected by ONT sequencing in infected T cells. Organization of HIV NL4-3 genome as well as position of SD and SA sites identified in Fig. 1 are indicated. Nomenclatures of introns and exons are according to [4, 5]. Only transcripts that were detected by ONT sequencing at least 5 times across infected CD4+ T cells are represented. Thick boxes correspond to retained exons and thin lines excised introns. Transcripts known or susceptible to encode the same viral proteins were grouped together and their ORF were color-coded (blue for Env/Vpu, green for Nef, yellow for Rev, pink for Tat, purple for Vif and red for Vpr). RNA species were named as indicated on the left side according to [4, 5, 10]. Putative new isoforms are indicated by black dots on the right side

To minimize possible artifacts arising during cDNA library preparation and analysis, only exon combinations present at least in 2 independent biological replicates and 5 times across the replicates were further considered as existing spliced isoforms [49]. This way, 53 exon combinations corresponding to 99% of the viral annotated reads in infected T cells were validated (Fig. 2, Additional file 4: Table S4 and Additional file 5: Figure S1). The same threshold was applied to infected and transfected HeLa samples, validating 45 and 54 different viral transcripts, respectively. It is noteworthy that a core of 36 transcripts was found in common for the three models, 24 h post-infection/transfection (Additional file 5: Figure S1). These isoforms were also detected in other HIV-1 transcriptomic assays [4, 5, 12].

Interestingly, our ONT assay was sensitive enough to detect transcripts expressed at very low levels such as Vif 1 and sORF 1, previously detected only by qPCR using specific pairs of primers [57]. In addition, we detected reads mapping to very short transcripts that could correspond to RNAs transcribed from unintegrated 2LTR circles [6, 58] (Fig. 2 and Additional file 4: Table S4). Transcription from the 2LTR circles was formerly detected by RT-PCR, but was never reported in high-throughput sequencing studies [59]. Here, we could identify transcripts starting at R, encompassing U5 and U3 and ending at R, therefore comprising the LTR–LTR junction. Visual inspection of 2LTR reads indicated that they were not PCR replicates (Additional file 6: Figure S2). Importantly, 2LTR RNA was detected only in infected HeLa and CD4+ T cells, but not in transfected conditions (Additional file 4: Table S4), reinforcing the hypothesis that this RNA is transcribed from non-integrated circular HIV-1 cDNA forms located in the nucleus of infected cells [59].

Finally, ONT sequencing allowed the detection of 14 potential new transcripts resulting from the use of rare or potential new splice sites. In particular, D1c spliced with different SA sites generating new putative isoforms such as Env/Vpu 18 and 19, Tat 9, Rev 13, Vif 4, Nef 12 and 16 (Fig. 2). We also detected 2 putative new Vif isoforms, Vif 3 and 5, resulting from splicing events involving the newly identified A1b SS, as well as isoforms involving D4a (Nef 13) or A5c (Nef 16). Alignments of reads assigned to these newly identified isoforms confirmed that they had different lengths and alignment coordinates, which suggests that they were not the result of PCR artifacts or misalignments (Additional file 6: Figure S2a) [60, 61]. Furthermore, although we could not confirm the existence of all of these new isoforms, specific primers overlapping several new splice junctions were designed and the PCR products were sequenced. We thus validated the existence of isoforms involving D1A5a (Env 17), D1cA5 (Env 19 and Nef 12), D1cA4b (Rev13), D1A1b (Vif 5), D1cA5c (Nef 16), D1cA3 (Tat 9) and D1cA1b (Vif 3) junctions in T lymphocytes (Additional file 6: Figure S2b). Altogether, our data indicate that between 4 and 11 different spliced isoforms can code for each viral protein in infected CD4+ T cells.

### Viral RNA isoform quantification

**Fig. 3:**
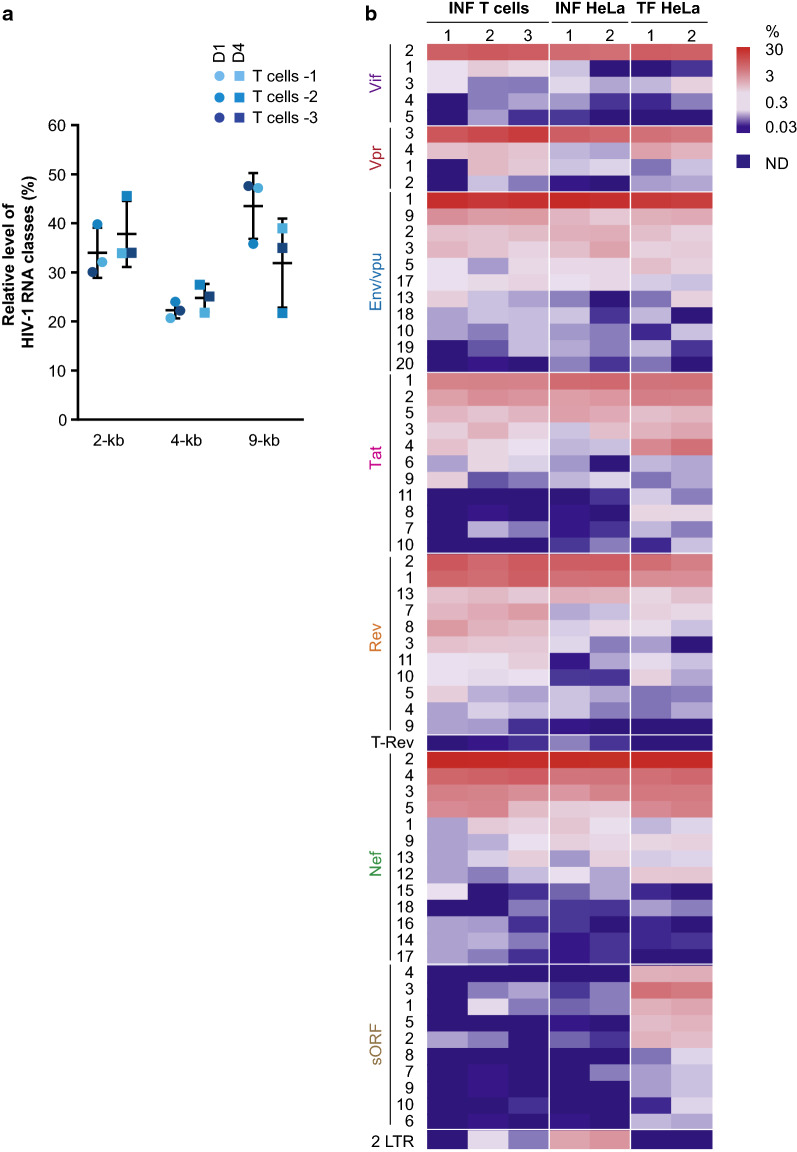
Quantification of viral isoforms produced in different HIV-1 expression models determined by ONT sequencing. **a** Relative abundance of viral RNA classes estimated either at D1 SS (dots) or at D4 SS (square) in infected CD4+ T cells from 3 different donors. Relative abundances of MS (2-kb), IS (4-kb) and US (9-kb) RNA classes were based on the number of reads harbouring or not a splice junction at D1 or D4 amongst all annotated reads as described in Additional file 8: Figure S3. Mean values and standard deviations are indicated. **b** Quantification of viral spliced isoforms expressed at 24 hpi in CD4+ T cells (INF T cells), HeLa cells (INF HeLa) or 24 h after transfection of HeLa cells (TF HeLa) according to ONT sequencing. Heatmap indicates the relative level of viral isoforms as a percentage of reads mapping to this particular transcript amongst reads mapping to all viral spliced transcripts. Only transcripts that were represented at least 5 times across replicates were considered. *ND* not detected

We next assessed the ability of ONT sequencing to quantify the relative abundance of viral isoforms. As for every cDNA sequencing assay, intrinsic biases linked to the polyA selection step, the reverse transcription, or the amplification step could be anticipated. Other biases specific to the ONT platform, such as sequencing preferences from the 5′ or the 3′ end of the cDNA through the pore or read lengths could also impact on read counts. The number of reads produced from the 3′-end (minus strand), or from the 5′-end (plus strand) proved to be very similar (Additional file 7: Table S5). In addition, the number of reads encompassing A7, D4 and D1 distributed all along the viral genome was comparable, suggesting that reverse transcription was relatively processive during the cDNA library preparations (Fig. 1, Additional file 7: Table S5). However, the mean read length of viral reads was comprised between 1058 and 1386 nucleotides (Additional file 1: Table S1) rendering the recovery of reads mapping the full-length US RNA unlikely, and more difficult for the 4-kb RNAs than for the 2-kb RNAs. To limit this bias, we estimated the relative abundance of spliced viral RNAs on the region surrounded by D1 and D4, which after splicing at D1 is the shortest region (< 1131 nucleotides long) of the genome allowing discrimination of all spliced isoforms without ambiguity (Additional file 8: Figure S3a). Based on the number of reads splicing at D1 with or without splicing at D4, ratios of 2- and 4-kb classes of RNA were calculated (Additional file 8: Figure S3b). Abundance of 9-kb RNA was estimated at both SS D1, by counting the number of reads that did not splice over the total number of reads at this position (Additional file 8: Figure S3c), and at SS D4, by taking into account the level of 2-kb and 4-kb RNAs previously determined (Additional file 8: Figure S3d). Both estimations were concordant and depending on the T cell donor, we evaluated that 34 to 38% of transcripts belonged to the 2-kb class whereas 22 to 25% are 4-kb class RNAs and 32 to 44% are 9-kb RNA (Fig. 3a). While the relative abundance of 2-kb and 4-kb RNAs were similar, we observed a limited bias due to the read length for the estimation of the 9-kb RNA. Nevertheless, this is in good agreement with previous works: the Stoltzfus laboratory predicted that about half of viral RNA remains US [9, 32], whereas by using a combination of PacBio sequencing to quantify similarly sized isoforms and Illumina sequencing to infer size class abundances, Sherrill-mix et al. estimated that US RNA represented 37.6% of viral transcipts abundance in infected cells [53].

The relative abundance of 53 transcripts was then quantified and we observed a remarkable correlation between T cell donors (r = 0.98) (Fig. 3b and Additional file 9: Figure S4). Consistent with previous reports, spliced transcripts belonging to the Nef (2-kb) and Env/Vpu (4-kb) families were the most expressed, representing more than 50% of all spliced transcripts, whereas Vif and Tat are the least abundant families (Fig. 3b) [4, 5]. Within each family, one or two transcripts were highly represented with other isoforms being expressed at much lesser levels. For instance, Nef 2, Env/Vpu 1 and Vpr 3 represented in average 62, 73 and 83% of their respective families, whereas Tat 1/Tat 2 and Rev 1/Rev 2 together corresponded to more than 60% of the transcripts of their respective families. We noted that whereas MS isoforms of Tat were predominant over IS, Vpr RNAs belonged mostly to the 4-kb class (Fig. 3b and Additional file 4: Table S4).

The relative quantifications of spliced isoforms in infected and transfected HeLa cells strongly correlated with that measured in infected T cells, indicating that splicing is similarly regulated across these different experimental settings (Fig. 3b and Additional file 10: Figure S5a, b and c). However, we noted that US RNA is less abundant in transfected cells compared to infected ones and MS RNAs were more abundant (Additional file 10: Figure S5a).

To further assess the ability of the ONT assay to quantify HIV-1 isoforms, we compared our data with results previously obtained by Purcell et al. using semi-quantitative RT-PCR and gel analysis of RNAs from HIV-1 NL4-3 infected PBMC [4]. For this, relative levels of viral isoforms obtained by ONT sequencing were calculated within the 2-kb and 4-kb classes of transcripts. Overall, ONT isoform quantifications strongly correlated with semi-quantitative RT-PCR quantifications (r = 0.97, p < 0.0001 for the 2-kb class and r = 0.81, p < 0.0001 for the 4-kb), confirming that the HIV-1 transcriptome profile generated by ONT sequencing recapitulates the classical RNA profile described in [4] (Additional file 11: Figure S6a and S6b). We noted some discrepancies for Vpr 3 and Vif 2 that appeared more expressed in our assay and Env/Vpu 1 that appeared less expressed than in [4] (Additional file 11: Figure S6b). This may be due to an overestimation of Vif 2 and Vpr 3 to the detriment of Env/Vpu 1 in our assay. However, Vif 2 and Vpr 3 are the longest isoforms and should therefore be the most difficult to reverse transcribe, to PCR-amplify and to detect in sequencing assays. It is also plausible that long isoforms such as Vif 2 and Vpr 3 were better processed by the new generation of RT used in this study and that these isoforms were more difficult to detect by gel analysis and therefore underestimated to the profit of Env/Vpu 1 in [4]. A higher proportion of D1A1 and D1A2 junctions than estimated in Purcell et al. were also reported by recent short read sequencing [12].

### Nanopore sequencing to study perturbation of HIV-1 alternative splicing

**Fig. 4:**
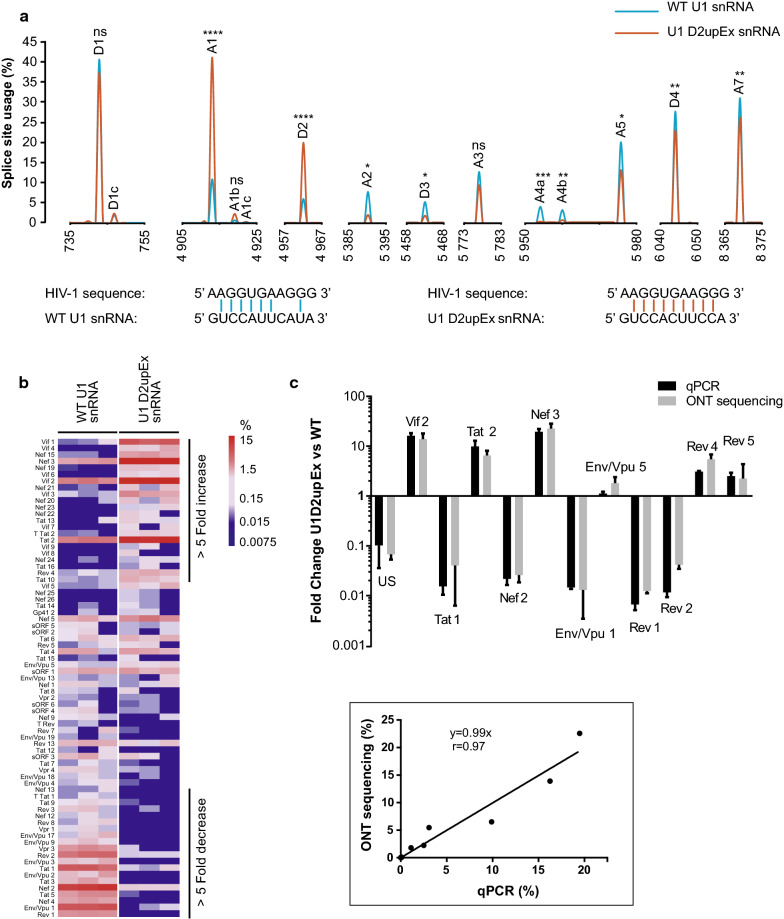
Characterization of HIV-1 RNA populations in HeLa cells expressing either wild-type or U1 D2upEx snRNA by ONT sequencing. HeLa cells were transfected with either the wild-type U1 snRNA or the modified U1 D2upEx snRNA enhancing SS D2 usage. 48 h later, cells were harvested, RNA was extracted and cDNA libraries were prepared. ONT sequencing and analyses were performed as described in Fig. 1. **a** Usage of SD and SA sites are expressed as a percentage of the occurrence of all SS within each condition. Data are presented as mean (n = 3). P values were calculated using an unpaired t-test (*p < 0.05, **p < 0.01, ***p < 0.001 and ****p < 0.0001). **b** Isoform expression levels were calculated for both wild-type and U1 D2upEx snRNA conditions. Heatmap represents the fold enrichment of isoforms expressed in U1 D2upEx snRNA expressing cells over wild-type U1 snRNA condition according to the coloured scale. For strongly downregulated RNAs that were not detectable anymore by ONT sequencing in U1D2upEx snRNA conditions, a correction of 0.0075% corresponding to the lowest  % calculated for the detection of a single read, was added. **c** Relative expression of 12 viral isoforms were measured by qPCR in WT and U1D2upEx snRNA expressing samples and fold changes were calculated as in **b**. US RNA levels were estimated at D1. Correlation curve was plotted using a linear regression model supplied by *Prism 7*. Pearson correlation coefficient r is indicated. p < 0.0001

We next examined the ability of ONT assay to follow changes in RNA levels when HIV-1 splicing was artificially perturbed using a modified U1 snRNA strategy [62, 63]. We followed the impact of either a wild-type (WT), or a modified spliceosomal U1 snRNA (U1 D2upEx snRNA) with increased affinity for the relatively weak viral SD site D2 on HIV-1 transcriptome (Fig. 4a) [32]. U1 D2upEx snRNA was previously shown to promote excessive splicing at D2 and to inhibit HIV-1 replication in T cells [32]. HeLa cells were thus co-transfected with NL4-3 provirus and either the control vector expressing WT U1 snRNA or the U1 D2upEx snRNA plasmid and subjected to ONT sequencing (Additional file 2: Table S2). To confirm the effect of U1 D2upEx snRNA expression, the relative SS usage was first examined (Fig. 4a). As expected D2 usage was increased by 3.3-fold in the presence of U1 D2upEx snRNA compared to WT U1 snRNA. Two potential new cryptic splice sites, D2c and D2d in the vicinity of D2 were identified exclusively in the presence of U1 D2upEx snRNA, while usage of the cryptic SS D2b was not impacted (Additional file 3: Table S3). Usage of the weak SD site D3 was further reduced by threefold, whereas D1 and D4 were modestly impacted. Definition of an exon relies on functional crosstalk between a 3′-SD site and the upstream 5′-SA site of the exon. This exon definition hypothesis predicts that binding of U1 snRNP to a downstream SD site can increase splicing at the upstream flanking SA site [64, 65]. Accordingly, usage of A1, upstream of D2, was increased by 3.8-fold (Fig. 4a). A1b, a newly identified SA site close to A1 was also upregulated (Additional file 3: Table S3) and another potential new SA site A1c was specifically detected in cells transfected with U1 D2upEx snRNA construct. Usage of A3 and A5 were either not, or only moderately down-regulated, whereas A2 and A4 were decreased (Fig. 4a).

The relative level of 74 transcripts was then assessed in WT and modified U1 snRNA conditions. Quantification data proved highly reproducible between experimental replicates (r > 0.95, Additional file 12: Figure S7) and highlighted major changes in viral RNA expression patterns upon U1 D2upEx snRNA expression (Fig. 4b). A 15-fold decrease of US RNA accompanied by a 1.6- and 1.25-fold increase in the level of IS and MS transcripts, respectively (Fig. 4b, c and data not shown), were observed in U1 D2upEx snRNA condition as compared to control. Among the 73 detectable spliced isoforms, 24 were exclusively detected in one or the other condition (Fig. 4b). In particular, U1 D2upEx snRNA induced expression of 5 new potential Vif isoforms and 4 new potential Nef isoforms resulting from increased usage of A1, A1b, A1c, D2 and D2b. The relative level of 9 other transcripts, including Nef 3, Tat 2 and Vif 2, were also increased by 5- to 33-fold in U1 D2upEx snRNA condition. In parallel, 16 RNAs, such as Env/Vpu 1, Nef 2 and Tat 5 had a 5- to 308-fold decreased levels in modified U1 snRNA condition. Fifteen isoforms, including all Vpr RNAs, were no longer detectable (Fig. 4b and Additional file 4: Table S4). Importantly, overexpression of the modified U1 snRNA did not alter morphology or growth parameters in our experimental conditions. Moreover, the level of 180,869 cellular transcripts were compared between WT and U1 D2upEx snRNA conditions and only 16 of them were significantly affected (p < 0.05, data not shown), illustrating the specificity of the U1 snRNA strategy to influence splicing of particular transcripts.

To assess the ability of ONT sequencing to accurately quantify changes in RNA expression, quantifications were compared to fold changes measured by qPCR using pairs of primers specific for 12 different transcripts (Fig. 4c). Although the limit of detection was better for qPCR than for ONT sequencing, results obtained by these two approaches strongly correlated (r = 0.97, p < 0.0001), confirming the ability of ONT sequencing to quantify the relative level of viral isoforms. It is noteworthy that the fold-change measured for Vif 2 correlated well with the one measured by qPCR, indicating that ONT quantification is not biased toward detection of this isoform.

### Quantitative representation of HIV-1 splicing program

**Fig. 5:**
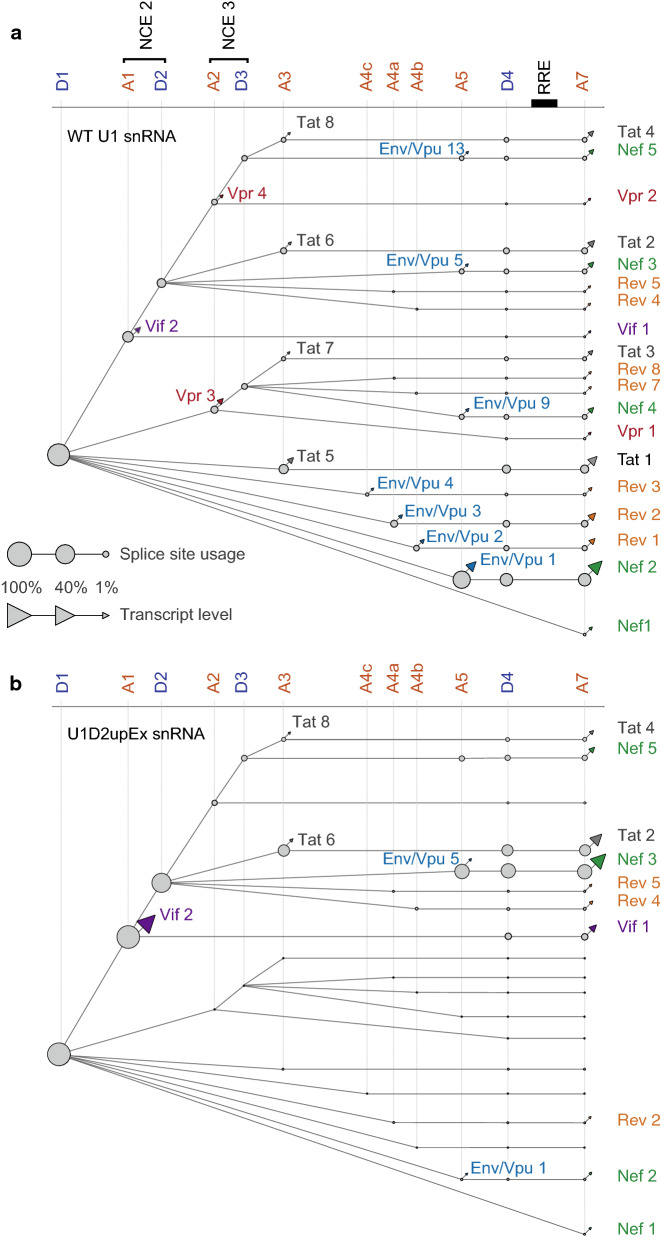
Effect of artificially enhanced splicing at D2 on HIV-1 alternative splicing regulation. Splice trees were drawn on *Cytoscape* based on ONT quantifications of SS usages and spliced isoform levels in **a** WT U1 snRNA and **b** U1D2upEx snRNA conditions. Only expression of transcripts that were found at least 5 times across the replicates and produced by usage of major SS were considered. Usage of SS are represented by nodes, lines symbolize splice junctions between SD and SA sites and the resulting isoforms are represented by triangles. Names of isoforms are indicated only if they could be detected in the condition. Sizes of nodes are function of relative SS usage and sizes of triangles are function of transcript levels as indicated by the scale. As D1 is present in all spliced isoforms, value of D1 was set at 100%. Non-coding exon (NCE) 2 and 3 are indicated

HIV-1 alternative splicing is post-transcriptional which implies that MS RNAs are generated from IS RNAs, which themselves are generated from the US precursor [66, 67]. Furthermore, HIV-1 alternative splicing was shown to proceed through a tight 5′ to 3′ order [68]. To get further insights into the effect of U1 D2upEx snRNA expression on the HIV-1 splicing program, quantification of splicing events was normalized to the total number of spliced transcripts produced in each condition and integrated into graphical representations that we called splice trees (Fig. 5). In these representations, nodes correspond to SD and SA sites and lines symbolize junctions between SS. Transcripts are represented by coloured triangles and the branches of the tree indicate the succession of splicing events from 5′ to 3′ leading to the production of each IS and MS isoforms. The size of the nodes and of the triangles is representative of SS usage and transcript level, respectively. For clarity, only major SS and major viral isoforms were taken into account. In WT condition, the most expressed isoforms within each family resulted generally from splicing between D1 and one of the SA (Env/Vpu 1, Nef 2, Rev 1, Rev 2, Tat 1, Vpr 3 and Vif 2) (Fig. 5a). A5 being the strongest SA site, Env/Vpu 1 as well as Nef 2 resulting from further splicing between D4 and A7, are the major spliced products confirming that splicing is in part driven by SA sites strength [9, 20]. Splicing to A3, A4 and A5 located in the 300 nucleotides upstream of D4 is often followed by additional splicing between D4 and A7, favouring production of MS RNAs (Nef 2, Rev 1, Rev 2 and Tat 1) [68]. In contrast, usage of A1 and A2 which are far upstream of D4, are usually followed by splicing with D2 and D3 but rarely with D4, favouring either the production of IS RNAs (Vif 2 and Vpr 3), or transcripts including NCE 2 and/or 3 (Fig. 5a).

As shown in Fig. 5b, U1 D2upEx snRNA expression induced a complete rewiring of splicing events to SA A1 and to the detriment of other branches of the splice tree (compare Fig. 5a, b): transcripts produced by splicing between D1 and A3, A4a and A5 were barely detectable and isoforms resulting from splicing between D1 and A4c and A4b were no longer detectable. Consequently, increased D2 usage led to an increased level of Vif 2 [32] and Vif 1. The U1 D2upEx snRNA was reported to enhance the inclusion of NCE 2 flanked by A1 in 5′ and D2 in 3′ [32]. This was reflected by an 8- and 30-fold increased production of Tat 2 and Nef 3, respectively, to the detriment of Tat 1 and Nef 2. However, increased inclusion of NCE 2 could not be extended to all isoforms: transcripts including both NCE 2 and 3 (Vpr 4, Vpr 2, Tat 8, Tat 4, Env 13 and Nef 5) were not upregulated or even downregulated in agreement with the apparent mutual exclusion of NCE 2 and NCE 3 (Fig. 5 and Additional file 4: Table S4) [57]. These results indicate that rather than increasing inclusion of NCE 2, improving splicing at D2 favours splicing events occurring at specific branches of the tree. The fact that MS RNAs of these branches (Tat 2 and Nef 3) are upregulated but not IS transcripts (Tat 6 and Env 5) even though they are produced by similar upstream splicing events illustrates how splicing to A3 or A5 SS is generally followed by further splicing between D4 and A7.

Altogether, these data demonstrate that the ONT assay is a sensitive method to quantify in detail splicing modulation and highlights the importance of considering the full extent of splicing events when studying splicing regulation.

### Dynamic of HIV-1 isoform levels in infected T cells by Nanopore sequencing

**Fig. 6:**
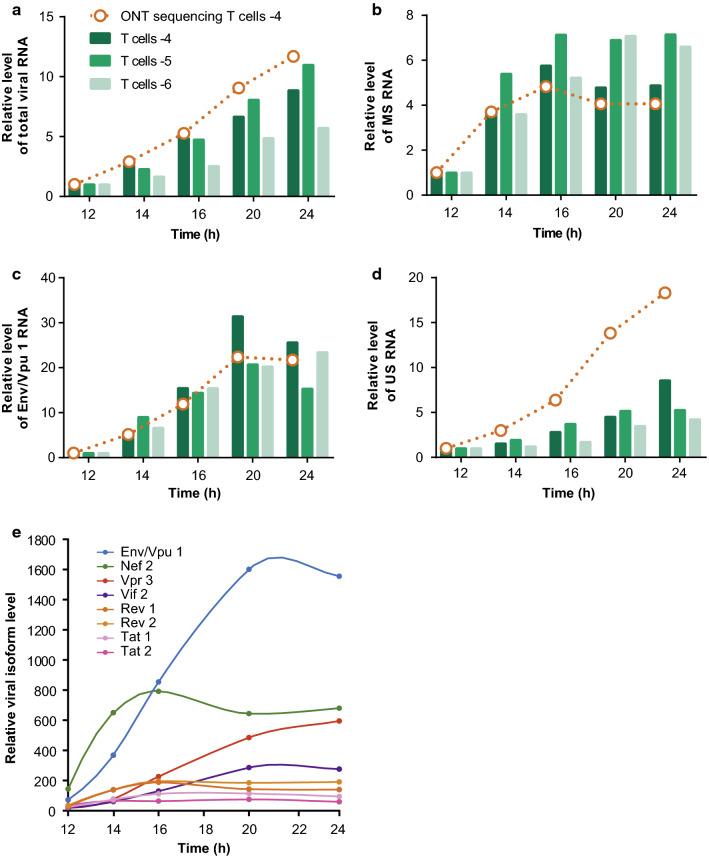
Relative abundance of viral transcripts expressed at early time points of T cell infection, determined by ONT sequencing. Primary CD4+ T cells were infected with HIV NL4-3 VSV-G pseudotyped virus and harvested at 12, 14, 16, 20 and 24 hpi for RNA extraction. Relative abundance of **a** Total, **b** MS, **c** Env/Vpu 1 and **d** US RNA was monitored by qPCR for three different donors using the ΔΔCq method and normalized with GAPDH and β-Actin as reference genes. ONT sequencing and mapping were performed on RNA extracted from donor 4 (orange lines). Read counts were normalized with *DESeq* *2* included in the Eoulsan’s pipeline. Abundance of viral RNA classes was calculated as in Fig. 3. All values were expressed as fold enrichment over the 12 h point. **e** Relative levels of the most abundant viral isoforms detected between 12 and 24 hpi were calculated as in Fig. 3, normalized using *DESeq* *2* and expressed as fold enrichment over the 12 h point

Finally, we took advantage of this assay to track the dynamic of splicing events regulating the production of HIV-1 isoforms at early times of infection. Activated CD4+ T cells from 3 different donors were infected at a low multiplicity of infection (MOI) to ensure that a majority of infected cells solely contained one virus per cell. Cells were then lysed at different time points between 12 h and 24 h post-infection (hpi) and RNA extracted. Using specific sets of primers, levels of total, US, Env/Vpu 1 (the major isoform of the IS class) and MS RNAs were monitored by qPCR. For donor 4, cDNA libraries were prepared from RNA extracted at 12, 14, 16, 20 and 24 hpi and sequenced on a MinION device (Fig. 6, Additional file 13: Table S6). All classes of transcripts were detected as soon as 12 h after infection and RNA levels were normalized to this time point (Fig. 6 and Additional file 14: Table S7). Depending on the donor, total HIV RNA increased 5 to 11-fold between 12 h and 24 hpi, likely reflecting the transactivation of HIV-1 transcription induced by Tat (Fig. 6a). However, concordant with the biphasic expression of early and late genes [6, 26, 27, 69], MS RNAs level increased rapidly between 12 and 14 h to reach a plateau at 16 hpi (Fig. 6b), whereas IS RNAs such as Env/Vpu 1 increased more progressively and until 20 hpi (Fig. 6c). Similar patterns were observed for the 3 infected T cell donors and the expression profiles of donor 4 defined by ONT sequencing closely matched those obtained by qPCR (Figs. 6a–c). In agreement with previous studies, US RNA increased continuously over the 24 h of infection (Fig. 6d). However, this increase appeared steeper when estimated by ONT sequencing than when quantified by qPCR, pointing to a possible overestimation of this particular class of transcript by long-read sequencing.

The kinetic of all the viral transcripts generated during infection of CD4+ T cells from donor 4 was then assessed by ONT sequencing. The major isoforms are presented in Fig. 6e and details of other isoforms are presented in Additional file 15: Figure S8. Whereas at 12 hpi Nef is the most expressed spliced isoform, Env/Vpu 1 increased by 15- to 30-fold to become the most abundant spliced viral RNA at 24 hpi. Vpr 3 and Vif 2 isoforms shared an identical IS profile and increased by 32- and 16-fold, respectively. Nef 2, Rev 1, Rev 2 and Tat 1 displayed the characteristic pattern of MS class with a rapid four to fivefold increase between 12 and 16 h and then reached a plateau. Even low expressed transcripts appeared to follow the specific pattern of their respective class (Additional file 15: Figure S8).

### HIV-1 alternative splicing program modulation during the early times of infection

**Fig. 7:**
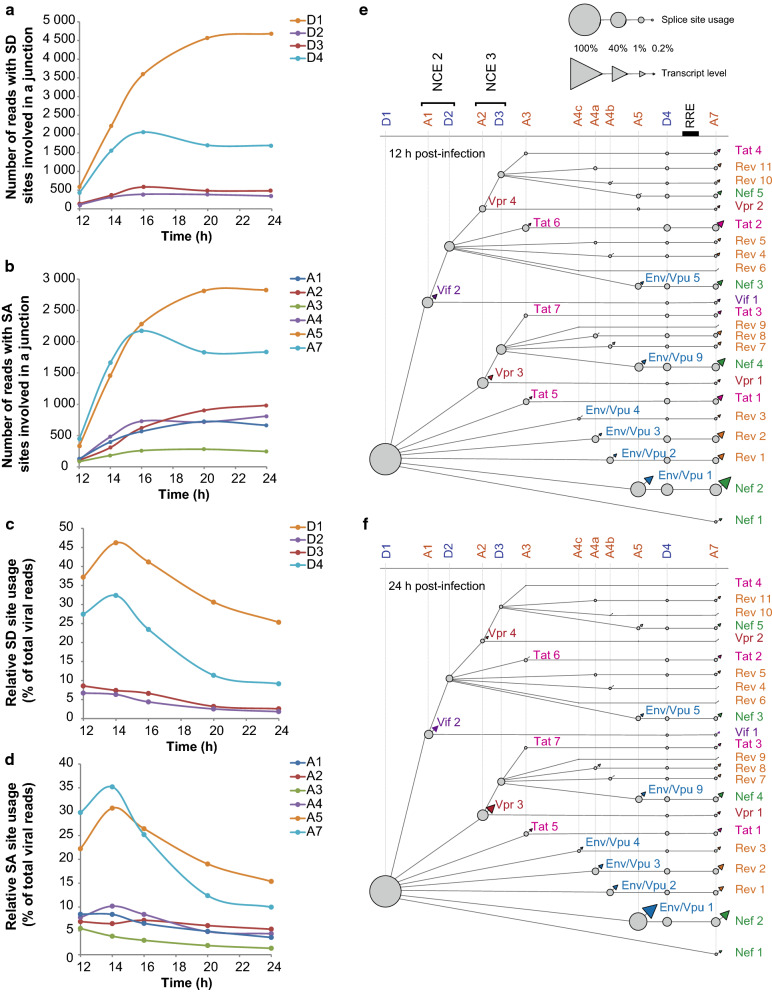
HIV-1 alternative splicing program in primary T cells at the early times of infection. Usage of SD (**a**) and SA (**b**) sites during a time course of infection of CD4+ T cells from donor 4 was based on the number of reads harbouring these particular SS involved in a splice junction and normalized with *DESeq2* included in the Eoulsan’s pipeline. Usage of SD (**c**) and SA (**d**) sites was then expressed as % of the total number of viral annotated reads at each time point. Splice tree representations of HIV-1 alternative splicing regulation at 12 (**e**) and 24 (**f**) hpi were drawn on *Cytoscape* as in Fig. 5 based on ONT quantification of SS usage and spliced isoform levels at each time point. Only transcripts that were found at least 5 times in a sample and produced by usage of major SS were considered. Size of nodes is function of relative SS usage and size of triangles is function of transcript level as indicated by the scale. As D1 is present in all spliced isoforms, value of D1 was set at 100%. Non-coding exons (NCE) 2 and 3 are indicated

Increased level of viral isoforms correlated with an increase involvement of SD and SA sites in splice junctions (Fig. 7a, b). However, differential abundance of viral isoforms during the course of infection is the result of a combination of transcription activation, RNA degradation, nuclear export and modulation of the splicing program. To further explore the mechanisms involved in the modulation of HIV-1 landscape during infection independently of the transcriptional effect, the relative SS usage was normalized to the total level of spliced products at each time point. A marked decrease of SD and SA sites engagement in splice junctions could then be observed starting from 14 hpi (Fig. 7c, d). This decrease mirrors the increase of 9-kb viral RNA over the time course of infection (Fig. 6d) and indicates a general repression of splicing events likely due to Rev-mediated export of US and IS isoforms. However, SD and SA sites seemed to follow different kinetics with for instance, the usage of D4 and A7 decreasing faster than D1 and A5 (Fig. 7c, d). Integration of SS usage and of spliced isoform quantification into splice trees at 12 h and 24 hpi enabled following the cascade of events and the interconnexions in the production of the different spliced isoforms throughout the infection (Fig. 7e, f).

**Fig. 8:**
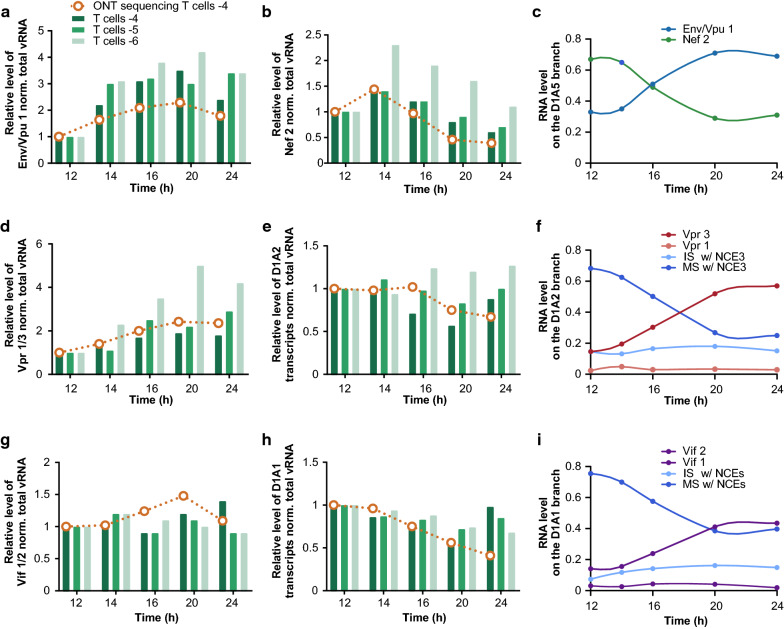
Detailed analysis of the balance of IS and MS isoforms during early times of CD4+ T cell infection. Relative level of IS RNA Env/Vpu 1 (**a**) and MS RNA Nef 2 (**b**) at different times post-infection of CD4+ T cells from three different donors were monitored by qPCR using the ΔΔCq method and normalized to the level of total viral RNA. Relative level of viral isoforms expressed in donor 4 determined by ONT sequencing is indicated in orange. ONT quantifications were based on the number of reads mapping to a particular isoform normalized by the total number of viral reads. **c** Dynamic expression of Env/Vpu 1 and Nef 2 monitored by ONT sequencing and normalized to the total number of transcripts belonging to the D1A5 branch of the tree. **d** Relative level of transcripts resulting from splicing between D1 and A2 without inclusion of NCE 3 (Vpr 1 and Vpr 3) was monitored by qPCR and ONT sequencing as in **a**. **e** Total number of transcripts generated by D1A2 splicing was monitored as in **a**. **f** ONT quantification of IS or MS isoforms generated by D1A2 splicing with (w/) or without NCE 3. Relative levels were normalized to the total level of D1A2 transcripts. **g** Relative level of transcripts resulting from splicing between D1 and A1 without inclusion of NCE 2 (Vif 1 and Vif 2) was monitored by qPCR and ONT sequencing as in **a**. **h** Total number of transcripts generated by D1A1 splicing was monitored as in **a**. **i** ONT quantification of IS or MS isoforms generated by D1A1 splicing with (w/) or without NCE 2. Relative levels were normalized to the total level of D1A1 transcripts

Production of IS RNAs requires both splicing between D1 and one SA site upstream of the ORF of interest and repression of downstream splicing events (Figs. 2 and 7e) [9, 20]. However, in what proportion the increase of IS RNAs is due to an increase of SA site usage or a decrease of downstream splicing events was not clear. ONT sequencing in donor 4 indicated that the 2.6-fold relative increase of Env/Vpu 1 between 12 and 24 hpi relied on a 1.2-fold upregulation of splicing from D1 to A5 and a 1.7-fold downregulation of D4A7 splicing, and consequently a decrease of Nef 2 production (Fig. 7e, f). Quantitative PCR on infected CD4+ T cells from 3 different donors confirmed the reciprocal increase of Env/Vpu 1 as the production of Nef 2 decreased (Fig. 8a, b) as seen by ONT sequencing in Fig. 8c. Similarly, downregulation of D4A7 junction correlated with an increase of Env/Vpu 2, Env/Vpu 3, Env/Vpu 4 and Tat 5 (Fig. 7e, f). The D4A7 junction appears therefore as the major regulator of isoform abundance resulting from splicing between D1 and A3, A4 and A5 SA sites between 12 and 24 h of infection. Nevertheless, D4A7 splicing rarely occurs after D1A1 (Vif 1) and D1A2 (Vpr 1). As shown in Fig. 7e, Vpr 3 was not regulated by an increase in usage of A2, nor by a decrease of the downstream D4A7 junction. Quantitative PCR confirmed that the two to fivefold relative increase in Vpr 3 (depending on the donor) was not related to an upregulation of D1A2 splicing (Fig. 8d, e). Instead, ONT sequencing revealed that Vpr 3 increased as splicing of D3 to downstream SA sites was downregulated, indicating that production of Vpr 3 was mainly regulated by exclusion of NCE 3 over the course of infection (compare Figs. 7e, f, 8f). Furthermore, detailed analysis of the D1A2 branch of the tree revealed that Vpr 3 upregulation was inversely correlated with the downregulation of MS isoforms containing NCE 3 (Nef 4 in particular) and not IS isoforms (Fig. 8f). Similarly, despite a relative decrease of D1A1 junction during infection, the level of Vif 2 seemed maintained throughout the infection to the detriment of subsequent spliced products between D2 and downstream SA sites rather than by a decrease of the D4A7 junction (Figs. 7e, f, 8g, h). When looking into the D1A1 branch of the tree, we observed that Vif 2 level was inversely correlated to the downregulation of MS isoforms containing NCE 2, in particular Nef 3 and Tat 2 (Fig. 8i). Altogether, these data strongly suggest that NCE 2 and NCE 3 serve as intermediates to regulate the temporal abundance of Vif 2 ans Vpr 3.

### Sequencing viral RNA in virions

Finally, we tested the possibility of sequencing RNAs present in viral particles. A library was prepared using RNA extracted from concentrated viral particles generated at 24 hpi from 10 × 10^6^ infected CD4+ T cells (donor 4). Although input RNA was too low to be quantified by bioanalyzer, 221,954 annotated viral reads were obtained. As expected, 99.95% of these reads mapped to full-length viral genomic RNA and only traces (78 annotated reads) corresponded to the most expressed transcripts (Nef 2, Env/Vpu 1, Nef 4, Vif 2 and Vpr 3) (Additional file 14: Table S7) [70]. This indicates that ONT sequencing can exploit low quantities of RNA present in purified viral particles.

## Discussion

To broaden our understanding of the regulation of HIV-1 transcriptome and the underlying alternative splicing events, we applied the recent development of ONT sequencing and quantified the steady-state level of all viral RNA isoforms in various infected cellular models (activated CD4+ T lymphocytes and Hela cells), in different production conditions (infection, transfection, in the presence of a wild-type or mutated spliceosomal machinery) and throughout a time course of primary CD4+ T cells HIV-1 infection.

With HIV-1 reads 7- to 10-times longer than in previous deep sequencing studies [5, 12], ONT sequencing now offers the opportunity to cover all the major splice junctions present in viral isoforms and thus, to resolve alternative isoforms containing even long-distant exons. Based on the splicing pattern, reads were mapped to viral spliced transcripts without bias inherent to the use of assembly algorithms and in total we identified 227 exon combinations including 175 new combinations in the 3 models of HIV-1 producing cells. However, one drawback of ONT sequencing is the high error rate as compared to deep sequencing technologies. *Minimap2* enables a global alignment of reads onto the annotated HIV-1 genome and discrimination of individual bases is not absolutely required to annotate spliced isoforms. Nevertheless, limitation in sequencing accuracy may suggest that some of the newly identified SS or splice junctions and the resulting isoforms could have been created by error. Furthermore, high error rate could complicate the resolution of SS that are very close to each other such as D1 and D1c which are only 4 nucleotides apart. Filters were thus applied during data analysis to limit this bias and only 53 mRNA isoforms, including 14 new viral isoforms in T lymphocytes were further considered in this analysis. Importantly, the existence of 2 of the 4 newly identified SS and 7 of the 9 newly identified splice junctions were confirmed, reinforcing the validity of our approach to identify new isoforms.

Another drawback of ONT sequencing is the relatively limited number of reads obtained, compared to short read sequencing drawing into question its sensibility to detect rare events. HIV-1 infection models complicate further the system, since less than 2% of the reads mapped to viral RNA diminishing the number of HIV-specific reads. We reassuringly detected all the isoforms previously reported in [4], most of the isoforms previously identified in other deep sequencing studies [5, 12], as well as several transcripts that were detected in other studies at very low levels such as Vif 1, sORF 1 and 2LTR RNA [57, 59], indicating that our assay provides a broad view of the complexity of HIV-1 RNA landscape. Overall, the low number of reads generated by ONT sequencing was compensated by read length giving unique information on the combination of splicing events.

The originality of our quantification assay is that reads were grouped based on their exon composition and then the number of reads mapping to each particular isoform were counted. Importantly, this method offers for the first time the opportunity to compare the overall abundance of viral RNAs belonging to all 3 classes in a single assay without skewing due to the use of primers specific for the 2-kb, 4-kb or 9-kb class during the PCR amplification. We acknowledge that, like for other cDNA sequencing technologies, bias inherent to the RT step remains during the procedure and our results suggest that US RNA level is possibly overestimated. ONT direct RNA sequencing would certainly allow to overcome this problem. However, we believe that our quantification method of spliced isoforms based on reads encompassing all splice junctions between D1 and D4 minimizes biases and is reliable: the high degree of reproducibility in the quantifications is to our opinion a first step to show the robustness of our assay (Additional file 12: Figure S7 and Additional file 9: Figure S4). Furthermore, we showed that ONT quantification of spliced isoforms highly correlated with qPCR quantifications when this confirmation was possible (Figs. 4, 6 and 8). Finally, ONT quantification data obtained within the MS and IS classes of RNA remarkably correlated with that obtained from PBMC infected with the same HIV-1 NL4-3 strain [4] and are in good agreement with other deep-sequencing studies [5, 12], validating our ONT sequencing assay to recapitulate the classical picture of HIV-1 transcriptome (Additional file 11: Figure S6).

Balanced production of viral isoforms during progression of infection is essential for HIV-1 replication [32, 71]. Early reports showed that MS RNAs were the first to be detected whereas IS and US RNAs were detectable later [6, 26]. A more recent work from Mohammadi et al. indicated that MS, IS and US RNAs were all detectable by qPCR from 15 hpi but that their respective peaks of expression were sequential [27]. Numerous studies have worked on the element regulating HIV alternative splicing, revealing how SS are modulated by a complex network of splice enhancers and silencers [7, 9, 20, 22]. Nevertheless, these studies were generally restricted to one or only few SS at a time. Two studies recently used deep sequencing to look at the differential effect of temperature on splice junctions [12] or at different times (18, 24 and 48 h) post-infection [5], providing a more dynamic view of the transcriptome regulation. However, with a mean read length of 249.5 nt for PacBio sequencing [5] and reads of 300 nt long for Illumina [12], these assays could only quantify relative abundance of splice junctions within MS, IS or US transcripts. Furthermore, the cascade of the early splicing events taking place during infection has never been investigated in detail. ONT sequencing allowed us to assess the dynamic changes of the viral transcriptome when one particular HIV-1 SS (D2) was artificially increased. We confirmed the changes in viral isoform levels reported in [32] and extended their study by examining in details how redirecting splicing impacts the entire splicing program. A key aspect of our analysis is that ONT sequencing enables us to determine how multiple splicing events are linked on a particular mRNA. Our splice tree models further provide a way to quantitatively represent the cascade of 5′ to 3′ splicing events and to analyze how splicing events influence other events at a transcriptome-wide level.

Finally, our assay proved sufficiently robust to follow the abundance of viral transcript classes over a time course of infection and to produce the most detailed picture of the splicing events occurring early after infection. It is important to highlight that our approach could only provide an approximation of the kinetics of viral isoforms rather than a precise timing. Indeed, we used VSV-G pseudotyped HIV-1 virus to infect primary CD4+ T cells wich results in faster entry through endocytosis possibly affecting the timeline of subsequent steps [72]. In addition, although spinoculation was used to concentrate viruses onto target cells, infection was not truly synchronized [73] and the timing may vary from one infected cell to another. Finally, as ONT sequencing is less sensitive than qPCR to detect very rare events, it could affect the time at which viral isoforms were detected and quantified. Nevertheless, the relative timing of viral isoform production during the course of infection correlates well with our qPCR data (Figs. 6 and 8) and with other studies. Our kinetics of HIV RNA abundance revealed a 4 h temporal shift in the maximum steady-state level of MS and IS RNAs over the time course of infection, while US RNA kept increasing at 24 h (Fig. 6). This is consistent with previous reports and with the 1.3 to 4.7 h delay estimated in expression of early and late gene reporters in infected MT4 cells [6, 26, 69]. Our data also revealed that while MS RNAs increased by around fivefold between 12 and 24 h of infection, IS increased by up to 30-fold. Nevertheless, when considering only spliced isoforms, changes in the splicing program between 12 h and 24 h of infection were more subtle (Figs. 7e, f and 8), highlighting the importance of the concomitant increase of transcription with the modulation of the splicing program in the expression of the viral transcriptome during infection.

Our data point out the production of IS transcripts, such as Env/Vpu 1–4 and Tat IS RNAs, is mainly upregulated by the repression of splicing events downstream of the ORF i.e. between D4 and A7, rather than by an increase of the corresponding SA A3, A4 or A5. Furthermore, NCE 2 and 3 represent an important means of regulating Vif 2 and Vpr 3 abundance during the natural course of infection. NCE 2 and 3 are highly conserved amongst HIV strains and have been proposed to interfere with RNA stability and gene expression, but their exact role in viral RNA production remained elusive [35, 71, 74]. It was suggested that SS D2 and D3 were involved in exon definition to activate the use of upstream A1 and A2, respectively [75–77]. Alternatively, the usage of D2 and D3 would maintain low levels of Vif 2 and Vpr 3 [12]. We observed that very early after infection, NCE 2 and NCE 3 were included, maintaining a very low level of Vif 2 and Vpr 3, respectively, and favouring further splicing between D4 and A7 to the profit of MS isoforms such as Tat 2, Nef 3, Nef 4 and several Rev isoforms. As the infection progresses, the splicing is redirected toward both a reduction of D4A7 junction and an exclusion of NCE 2 and 3 resulting in an increased production of Vif 2 and Vpr 3. Importantly, the presence of these NCE can generate more than 24 different isoforms (Figs. 2 and 7e). Whether this redundancy of templates coding for the same set of viral proteins is necessary for HIV-1 replication, or whether the variety of isoforms is generated as by-products by inefficient and noisy alternative splicing is a matter of debate [9, 35, 74]. Our data suggest that they are generated as part of the mechanisms involved in the fine tuning of Vif 2 and Vpr 3 proteins expression needed over the time course of infection.

## Conclusion

Our study shows that ONT sequencing, in combination with a straightforward in-house pipeline analysis allows to recapitulate the classical map of HIV-1 transcriptome expressed in infected CD4+ T cells in a fast, cost-effective and highly reproducible manner. Furthermore, due to an unprecedented viral read length, this assay gives access to a new kind of information such as the combination of distant splicing events. We propose an original graphical view based on big data to browse the transcriptional landscape of HIV-1 and to better understand the link between the production of transcripts and the splicing regulation. Integration of quantification data into splice tree representations allowed to accurately capture the complex remodelling of HIV-1 alternative splicing program when HIV-1 splicing was artificially perturbed. Importantly, we provide for the first time a full picture of the cascade of splicing events and the crosstalk between splice sites that shapes viral RNA landscape during the early steps of infection in primary CD4+ T cells.

We believe that this assay could be a powerful tool to clarify the role of *cis*-acting RNA elements and proteins that regulate splicing in HIV-1 infection, as well as the importance of structures surrounding these SS, and could help decipher the splicing code driving HIV-1 alternative splicing [1, 9, 13].

## Methods

### Plasmids

The HIV-1 proviral clone pNL4-3 was developed by M. Martin through the NIH AIDS Reagent Program [78]. Plasmids pUC13-U1 and pU1D2upEx were a kind gift from C.M. Stoltzfus [32]. They were respectively used to express the wild-type U1 snRNA and the modified U1 D2upEx snRNA with extended match to the 5′ splice site D2 of HIV-1.

### Isolation of primary CD4+ T cells and cell culture

CD4+ T cells were extracted from healthy donor blood (Etablissement Francais du Sang, EFS) with continuous-flow centrifugation leukophoresis product using density centrifugation on a Ficoll-Paque Plus gradient (GE HealthCare Life Science). CD4+ lymphocytes were purified from peripheral blood mononuclear cells (PBMCs) by negative selection with CD4+ T Cell isolation kit (Miltenyi Biotec) and activated with an equal amount of the provided biotinylated antibodies against CD2, CD3 and CD28 loaded on MACSiBeads Particles (Beads-to-cell ratio 1:2) (T cell Activation/Expansion kit; Miltenyi Biotec) for 3 days. CD4+ lymphocytes were cultured in RPMI medium 1640 (1X) + GlutaMAX (Gibco, Life Technologies) supplemented with 10% FBS (Gibco, Life Technologies), penicillin (100 U/ml), streptomycin (100 µg/ml), amphotericin B (0.25 µg/ml) (Gibco, Life Technologies), and stimulated with 30 U/ml IL-2 (Sigma Aldrich) (37 °C, 5% CO_2_). Activated cells were characterized by flow cytometry with CD25-APC (BD Pharmingen), CD69-PE (BD Pharmingen) HLA-DR-PERCP-Cy5.5 (BD Bioscience) and KI-67-FITC (DAKO).

HeLa and HEK293T cells were cultivated at 5% CO_2_ and 37 °C in DMEM (Life technologies) supplemented with 10% FBS (Gibco, Life technologies).

### Virus stock production

Stocks of VSV-G pseudotyped NL4-3 HIV-1 virus were produced by transfecting 4 × 10^6^ HEK293T cells with 3 µg of pNL4-3 and 1 µg of pMD.G (VSV-G) expression vectors using polyethylenimine (PEI) (Polysciences). Virus containing supernatants were harvested 48 h later, filtered and purified by ultracentrifugation on a sucrose cushion. Prior to infection, viral stocks were treated with 100 U/ml of DNase I (Roche Applied Science) for 1 h at 37 °C in the presence of 10 mM MgCl_2_. Infectious viral titers were assessed by infection of HeLa cells or activated CD4+ T cells with serial dilutions. Twenty-four hpi, percentage of infected cells was determined by FACS analysis by following intracellular capsid (anti-CAp24 antibody, KC57-FITC, Beckman Coulter).

### Co-transfection and infection of cells

Hela cells were plated into 100 mm diameter tissue culture dishes (Corning) in order to reach approximately 80% confluence at the transfection or infection time. To compare HIV-1 transcriptome in different models, HeLa cells were either infected with VSV-G pseudotyped NL4-3 virus at an MOI of 1, or transfected with 6 µg of NL4-3 plasmid using Lipofectamine LTX (Thermofisher) according to manufacturer instructions. Twenty-four hour post-infection or transfection, cells were harvested, washed with PBS and RNA was extracted. To artificially perturb HIV-1 splicing, HeLa cells were transfected with a mix of pNL4-3 (6 µg) and either pUC13-U1 or pU1D2upEx (4 µg) and harvested 48 h later for RNA extraction.

To analyze viral RNA population in T cells, 10 × 10^6^ activated CD4+ T cells were spinoculated with VSV-G pseudotyped NL4-3 viruses at an MOI of 1 for 2 h at 1300 g, 37 °C. After spinoculation, cells were washed three times in medium, resuspended at 1.5 × 10^6^ cells/ml of complete medium and IL-2 (30 U/µl) and incubated at 37 °C. Infected cells were quantified by flow cytometry 24 hpi using an anti-CAp24 antibody (KC57-FITC, Beckman Coulter) and showed a 57 to 81% of infected cells. To analyze viral RNA production during a time course of infection, 30 × 10^6^ cells were spinoculated with VSV-G pseudotyped NL4-3 viruses at an MOI of 0.2 for 2 h at room temperature as described in [79]. After 3 washes, cells were resuspended at 1.5 × 10^6^ cells/ml, aliquoted at 6 × 10^6^ cells per time point and further incubated at 37 °C. Cells were collected at different time points between 12 h and 24 hpi and viruses containing supernatants were collected at 24 hpi, filtered and pelleted through a 20% sucrose cushion by ultracentrifugation at 150,000×*g* for 90 min.

### RNA extraction and DNase treatment

Total RNA from viruses, infected or transfected cells was extracted with RNeasy Mini Kit (Qiagen). On-column DNase treatment (Qiagen) was performed following provider’s instructions. For long-read sequencing, samples were treated with a second DNase digestion using TURBO DNA-free™ kit (Ambion). The purity and quantity of RNA samples were checked with a NanoDrop 1000 spectrophotometer (Nanodrop Technologies).

### Relative quantification of HIV-1 transcripts by qPCR

Purified RNAs were reverse transcribed using the High-Capacity cDNA Reverse Transcription Kit (Applied Biosystems) and random primers to avoid bias against long transcripts. Quantification was performed by real-time PCR using LightCycler 480 SYBR Green I Master (Roche) and primer pairs specific for each viral transcript (Additional file 16: Table S8). Total viral RNA primers were used to normalize the relative level of each viral isoform, calculated by the ΔΔC_q_ method. The relative level of viral RNA classes at early points after infection was normalized using primers for two reference genes (GAPDH and β-Actin, Additional file 16: Table S8).

### Long-read sequencing with MinION instrument from Oxford Nanopore Technologies

Library preparation was done using SQK-LSK108 following manufacturer’s protocol (1D PCR Barcoding cDNA; ONT) optimized for cDNA sequencing. Briefly, 100 ng of total RNAs was reverse transcribed for each sample (50 °C 10 min, 42 °C 10 min, 80 °C 10 min), using custom polyT-VN and strand switching primers, with SuperScript IV (Life Technologies; 18090010). Use of custom polyT adaptors presented the advantage to limit cDNA amplification to only full polyadenylated transcripts. Library protocol including strand switching mechanism at the 5′ end of RNA template favoured full length cDNA synthesis. The reaction was purified with 0.7X Agencourt Ampure XP beads. A quarter of the purified RT product was taken into PCR for 18 cycles of amplification (95 °C 15 s, 62 °C 15 s, 65 °C 17 min) and barcodes addition (Barcoded primers form EXP-PBC001, ONT). Double stranded cDNAs were purified as above, quantified and their sizes were checked. Samples were multiplexed in equimolar quantities to obtain 1 µg of cDNA. The pool was end-repaired and dA-Tailed using the NEBNext End repair/dA-tailing Module (New England BioLabs E7546) and purified with 1X Agencourt beads. Adapter ligation was performed at room temperature for 10 min, with Adapter Mix (AMX, ONT) and Blunt/TA Ligase Master Mix (New England BioLabs M0367). After a final 1X clean-up and washing of the beads with Adapter Binding Buffer (ABB, ONT), the library was eluted in 15 µl Elution buffer. A quantity of 300–350 ng of cDNA was loaded on the flowcell (R9.4 or R9.4.1) after priming it, according to the manufacturer’s protocol. Sequencing was performed with the standard 48 h sequencing protocol run on the MinION MkIB, using the *MinKNOW* software (v 1.10.23 and 1.11.5). Base-calling from read event data was performed by *Albacore* (v 2.1.10 and 2.2.7).

To analyze viral RNA produced during a time course of infection, the protocol was upgraded as followed: 10 ng of total RNA were amplified and converted to cDNA using SMART-Seq v4 Ultra Low Input RNA kit (Clontech). Afterwards an average of 14 fmol of amplified cDNA was used to prepare library following SQK-PBK004 kit (PCR Barcoding kit; ONT). After the PCR adaptater ligation, a 0.6X Agencourt Ampure XP beads clean-up was optimised and 2 fmol of the purified product was taken into PCR for amplification and barcodes addition with a 17 min elongation at each 18 cycles. Samples were multiplexed in equimolar quantities to obtain 100 fmol of cDNA and the rapid adapter ligation step was performed. All the prepared library was used for loading on an R9.4/R9.4.1 flowcell according to the manufacturer’s protocol. Sequencing was performed with the standard 48 h sequencing protocol run on the MinION MkIB, using the *MinKNOW* software (v 3.3.2) and 7.5 million reads were obtained for the run. A mean of 1.1 ± 0.5 million passing ONT quality filter reads was obtained for each of the 5 samples. Base-calling from read event data was performed by *Guppy* (v 3.1.5).

### Alignment with *Minimap2*

The analyses were performed using a part of the Eoulsan pipeline [80], including read filtering, mapping with *Minimap2* (v 2.10) [50] (specific command line: −x splice −k 14) and alignment filtering. Before mapping, polyN read tails were trimmed. Reads were then aligned against a merged version of the *Homo sapiens* genome (from Ensembl version 91—human GRCh38 assembly) and the HIV genome AF324493.2 from the NCBI. Unmapped reads, alignments with a quality lower than 1 and alignments from reads matching more than once on the reference genome were discarded using *SAMtools* [81]. Alignment files were converted into BAM format. Sashimi plot was obtained using Integrative Genomics Viewer *IGV* (v 2.6.3) [82] on alignment files from infected T cell samples.

### Annotation of HIV-1 transcripts quantification and splice tree design

Reads were screened for potential SD and SA sites by identifying exon start/end positions from BED files analyses. Reads presenting the same combination of splice junction and exonic sequences were grouped together and counted. Only potential SD and SA sites found in reads ≥ 5 copies inside replicate conditions were considered in the rest of the analysis. If the positions of potential SD/SA sites were unknown, only those implicated in the canonical GT-AG splice site pairs were annotated.

Transcript isoforms were annotated according to the exon combination present in each read ≥ 5 copies inside replicate conditions. Annotation was performed according to convention established in [4, 5, 10] or, for potential new spliced isoforms, to the open reading frame (ORF) encountered in the read. For spliced transcripts, only complete sequencing reads (CSR) encompassing both junctions involving D1 (or D1c) and D4 (or D4a or D4b) SS were considered and assigned without ambiguity to a specific isoform.

To validate new and rare transcripts, total RNA from infected CD4+ T cells were reverse transcribed using the High-Capacity cDNA Reverse Transcription Kit (Applied Biosystems) and random primers and specific primers (Additional file 16: Table S8) were used to amplify new junctions. Purified PCR products were sequenced by Sanger sequencing.

Relative levels of spliced viral isoforms were quantified by dividing the number of CSR assigned to this particular isoform by the total number of CSR (Additional file 8: Figure S3). The abundance of MS and IS RNAs were calculated by dividing the total number of reads belonging to each class by the total number of CSR. Relative level of 9-kb transcripts was estimated at both D1 and D4 SS by dividing the number of reads, including partial sequencing reads encompassing D1 or D4 without splicing, by the total number of reads spanning this SS (Additional file 8: Figure S3).

For kinetics of donor 4, the read count of all samples were normalized using *DESeq* *2* (v 1.8.1) [83].

Splice trees were built by integrating quantification data of spliced transcripts normalized to the total level of spliced transcripts into *Cytoscape* (v 3.6.1) [84]. For clarity only transcripts ≥ 5 copies and using the main splice sites were represented. Usage of D1 was set at 100%.

## Supplementary information

**Additional file 1: Table S1.** Read mapping statistics of HIV-1 infected T cell samples, sequenced with ONT device. Activated CD4+ T cells from 3 different donors were infected with NL4-3 VSV-G pseudotyped virus, harvested 24 h later and RNA was extracted. cDNA libraries were prepared and sequenced using MinION device from ONT. Raw reads from 3 different CD4+ T cell samples were mapped onto the human and the  HIV-1 NL4-3 genome using Minimap2.

**Additional file 2: Table S2.** Read mapping statistics of HIV-1 expressing HeLa cell samples, sequenced with ONT device. ONT sequencing reads were mapped to both the human and HIV NL4-3 genomes, using *Minimap2*. NI: non-infected HeLa cells; INF: infected HeLa cells; TF: transfected HeLa cells; WT U1 HeLa: wild type U1 snRNA co-transfected HeLa cells; U1 D2upEx HeLa: modified U1 D2upEx snRNA co-transfected HeLa cells.

**Additional file 3: Table S3.** NL4-3 splice site counts in HIV-1 expressing samples. Reads overlapping potential SD/SA sites were counted and pooled for each HIV-1 expressing sample (infected T cells; Transfected HeLa cells; Infected HeLa cells; WT U1 HeLa: HeLa cells co-transfected with wild-type U1 snRNA and NL4-3 provirus; U1 D2upEx HeLa: HeLa cells co-transfected with modified U1D2upEx snRNA and NL4-3 provirus). Splice sites observed at least 5 times in a same splice junction of a transcript are highlighted in grey. Known SA/SD were named according to the established classification. Putative splice sites were considered as new only if they were represented in a splice junction of a transcript represented at least 5 times and if they were involved in a consensus splice junction (GT-AG).

**Additional file 4: Table S4.** HIV-1 transcripts identified in the different cellular models. Reads mapping to each isoform were counted and pooled for each HIV-1 expression model. Isoforms represented by ≥5 reads in T cells were highlighted in grey. The class of transcripts, the splice junctions involved and the study where they were first identified are indicated. New exon combinations represented by less than 5 reads were not named (ND: not defined). N: New isoform identified in this study.

**Additional file 5: Figure S1.** Viral isoform levels in different models of HIV-1 expressing cells. (a) Exon combinations identified by ONT sequencing in infected T cells (INF T cells), transfected (TF HeLa) or infected (INF HeLa) HeLa cells. (b) Exon combinations ≥5 copies amongst replicates and considered as existing viral isoforms in the rest of the analysis.

**Additional file 6: Figure S2.** Identification of new and rare transcripts involving SS D1c, D4a, A1b, A5a and A5c and LTR2 RNA in HIV-1 infected T cell samples. (a) IGV screenshots of ONT read alignments of new and rare transcripts described in the Additional file 4: Table S4. (b) New and rare transcripts were reverse transcribed and amplified using specific primers (Additional file 16: Table S8). DNA sequencing chromatograms of confirmed junctions are presented.

**Additional file 7: Table S5.** Details on read counts in infected CD4+ T cells.

**Additional file 8: Figure S3.** Estimation of the relative abundance of HIV-1 mRNA size classes using ONT sequencing. (a) Schematic representation of HIV-1 unspliced (US, 9-kb), incompletely spliced (IS, 4-kb) and multiply-spliced (MS, 2-kb) classes of HIV-1 RNA. SS used to calculate the levels of HIV-1 classes are indicated. Excised introns are represented as dotted lines and conserved exons as filled lines. Complete sequencing reads (CSR) corresponding to annotated reads starting before D1, ending after D4 and harbouring a least one splice junction involving D1 are indicated. (b) Relative quantification of MS and IS isoforms were calculated by dividing the number of CSR including (2-kb) or not (4-kb) a splice junction at D4 by the total number of CSR. (c) Relative quantification of 9-kb and spliced RNAs at D1 were estimated by counting the number of reads splicing (2-kb+4-kb) or not (9-kb) at D1 by the total number of reads passing through D1. (d) Relative quantification of 2-kb and 9-kb or 4-kb RNAs were estimated by counting the number of reads splicing (2-kb) or not (4-kb+9-kb) at D4 by the total number of reads passing through D4. Relative level of 9-kb, 4-kb and 2-kb RNAs in Fig. 3 was estimated by integrating the levels of each class determined in (b), (c) and (d).

**Additional file 9: Figure S4.** Correlation of viral isoform abundances quantified by ONT sequencing between T cell replicates. The relative abundances of HIV-1 spliced RNAs were calculated as a % of the total number of spliced viral RNAs. Results were compared between infected T cell samples obtained from 3 different donors using a linear regression model supplied by *Prism 7*: (a) donor 1 vs donor 2; (b) donor 1 vs donor 3; (c) donor 2 vs donor 3. Pearson correlation coefficients r are indicated. p<0.0001.

**Additional file 10: Figure S5.** Relative abundances of viral isoforms in infected and transfected HeLa cells. (a) Relative levels of viral RNA classes in HeLa cell were estimated as described for infected T cells (Fig. 3 and Additional file 8: Fig. S3). (b) Correlation of viral isoform abundances expressed in infected T cells versus transfected HeLa cells according to ONT sequencing. (c) Correlation of viral isoform abundances expressed in infected T cells versus infected HeLa cells according to ONT sequencing. Pearson correlation coefficients r are indicated. p<0.0001.

**Additional file 11: Figure S6.** Comparison of relative viral RNA abundances assessed by ONT sequencing and semi-quantitative PCR and gel analysis. Relative abundances of viral isoforms determined by ONT sequencing in Fig. 3 were expressed as a % of the total number of transcripts within either (a) MS RNAs or (b) IS RNAs. Results were compared with quantifications obtained in NL4-3 HIV-1 spreading infection of PBMC by semi-quantitative RT-PCR and gel analysis in [4]. Correlation curves using a linear regression model supplied by *Prism 7* are shown and Pearson correlation coefficients r are indicated. p<0.0001.

**Additional file 12: Figure S7.** Correlation of viral isoform abundances quantified by ONT sequencing between biological replicates of HeLa cells expressing either wild-type or U1 D2upEx snRNA. Relative abundances of HIV-1 spliced RNAs were calculated as a % of the total number of viral annotated reads. Results were compared using a linear regression model supplied by *Prism 7* between WT U1 HeLa cells samples: (a) sample 1 vs sample 2; (b) sample 1 vs sample 3; (c) sample 2 vs sample 3, and between U1 D2upEx HeLa samples : (d) sample 1 vs sample 2; (e) sample 1 vs sample 3; (f) sample 2 vs sample 3. Pearson correlation coefficients r are indicated. p<0.0001.

**Additional file 13: Table S6.** ONT read mapping statistics of T cell sample infected with HIV-1 between 12 h and 24 h. Primary CD4+ T cells from donor 4 were infected with VSV-G pseudotyped NL4-3 virus, RNA was extracted at 12, 14, 16, 20, 24 hpi, cDNA libraries were prepared and sequenced using ONT device. ONT sequencing reads were mapped to both the human and HIV NL4-3 genomes, using *Minimap2*.

**Additional file 14: Table S7.** Viral transcripts identified in HIV-1 infected T cells between 12 h and 24 h of infection and in viral particles. ONT sequencing reads mapping to each isoform expressed in infected CD4+ T cells from donor 4 at each time point, as well as reads produced from ONT sequencing of viral particles produced at 24 hpi were counted. The number of reads, the class of transcripts and the splice junctions involved are indicated. New exon combinations represented by less than 5 reads were not annotated (ND: not defined). N: New isoform identified in this study.

**Additional file 15: Figure S8.** Relative abundance of viral transcripts expressed at early time points of HIV-1 infection in CD4+ T cells, determined by ONT sequencing. Abundance of all viral transcripts expressed in CD4+ T cells from donor 4 between 12 h and 24 hpi was determined as in Fig. 6. Each panel corresponds to a family of transcripts: (a) Nef, (b) Env/Vpu, (c) Vpr, (d) Rev, (e) Tat and (f) Vif.

**Additional file 16: Table S8.** List of primers used in this study.

## Data Availability

The datasets generated and analyzed during the current study are available in the GEO repository (https://www.ncbi.nlm.nih.gov/geo/) under the accession number GSE138425.

## Abbreviations

HIV-1: Human immunodeficiency virus type 1
VSV-G: Vesicular stomatitis virus glycoprotein
MOI: Multiplicity of infection
pre-mRNA: Pre-messenger RNA
ONT sequencing: Oxford Nanopore Technologies sequencing
qPCR: Quantitative polymerase chain reaction
SS: Splice site
snRNA: Small nuclear RNA
SD site: Splice donor site
SA site: Splice acceptor site
kb: Kilobase
US: Unspliced
IS: Incompletely spliced
MS: Multiply spliced
NCE: Non-coding exon
RRE: Rev-response element
RT-PCR: Reverse transcriptase-PCR
NGS: Next generation sequencing
WT: Wild type
r: Correlation coefficient
hpi: Hours post-infection
