# Supplementary material for: Dynamic nanopore long-read sequencing analysis of HIV-1 splicing events during the early steps of infection
Source: Retrovirology. 2020 Aug 17;17:25. doi: 10.1186/s12977-020-00533-1 (PMC7433067; doi:10.1186/s12977-020-00533-1)
Supplement: Supplementary file 5 — Additional file 5: Figure S1. Viral isoform levels in different models of HIV-1 expressing cells. (a) Exon combinations identified by ONT sequencing in infected T cells (INF T cells), transfected (TF HeLa) or infected (INF HeLa) HeLa cells. (b) Exon combinations ≥5 copies amongst replicates and considered as existing viral isoforms in the rest of the analysis. [file 12977_2020_533_MOESM5_ESM.pptx]

## Slide 1
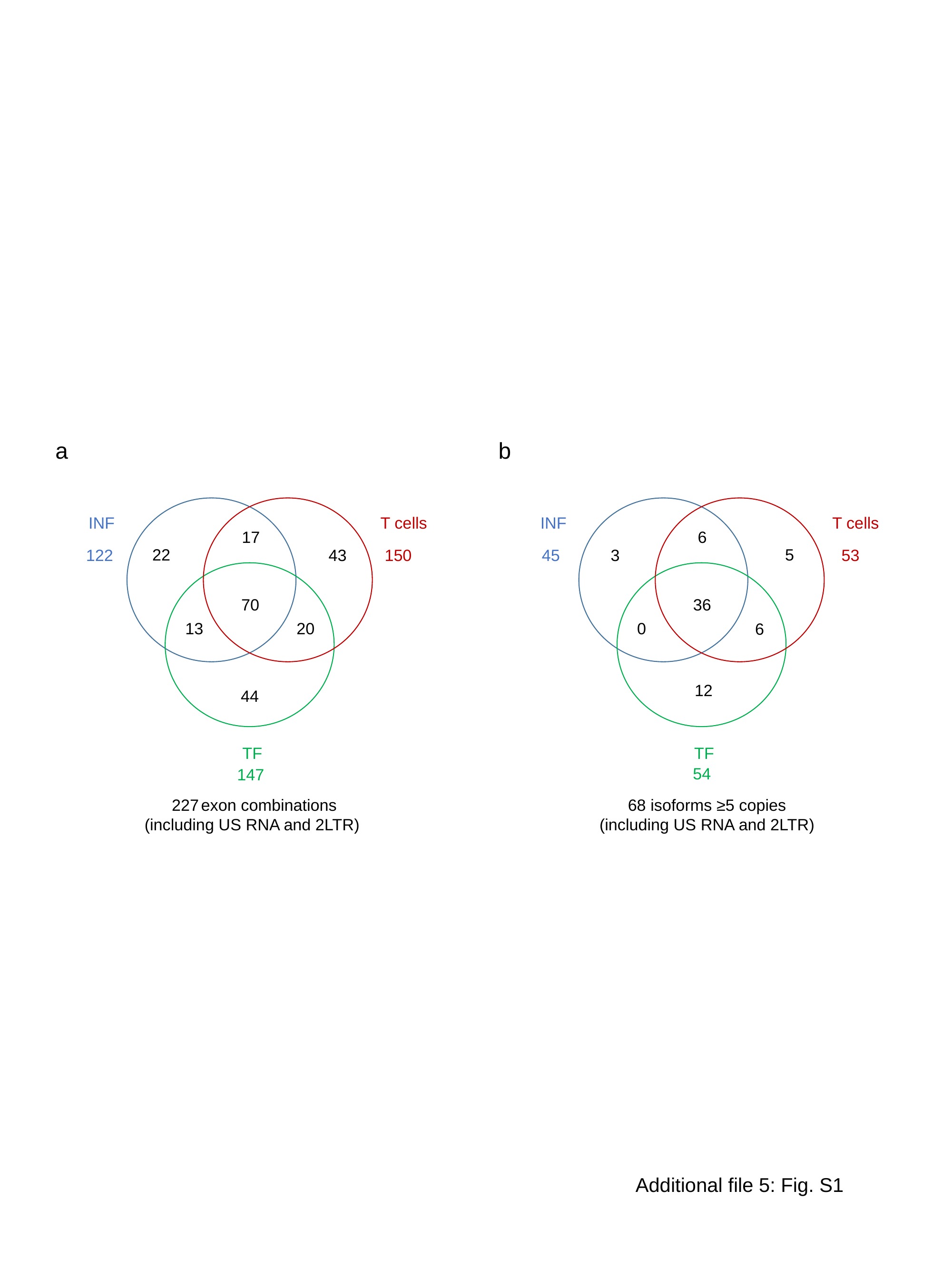

a
b
INF
T cells
6
45
53
36
0
6
TF
54
INF
T cells
17
122
150
70
13
20
TF
147
5
22
43
3
12
44
exon combinations
(including US RNA and 2LTR)
68 isoforms ≥5 copies
(including US RNA and 2LTR)
Additional file 5: Fig. S1
