# Supplementary figures and images for: Dynamic nanopore long-read sequencing analysis of HIV-1 splicing events during the early steps of infection
Source: Retrovirology. 2020 Aug 17;17:25. doi: 10.1186/s12977-020-00533-1 (PMC7433067; doi:10.1186/s12977-020-00533-1)

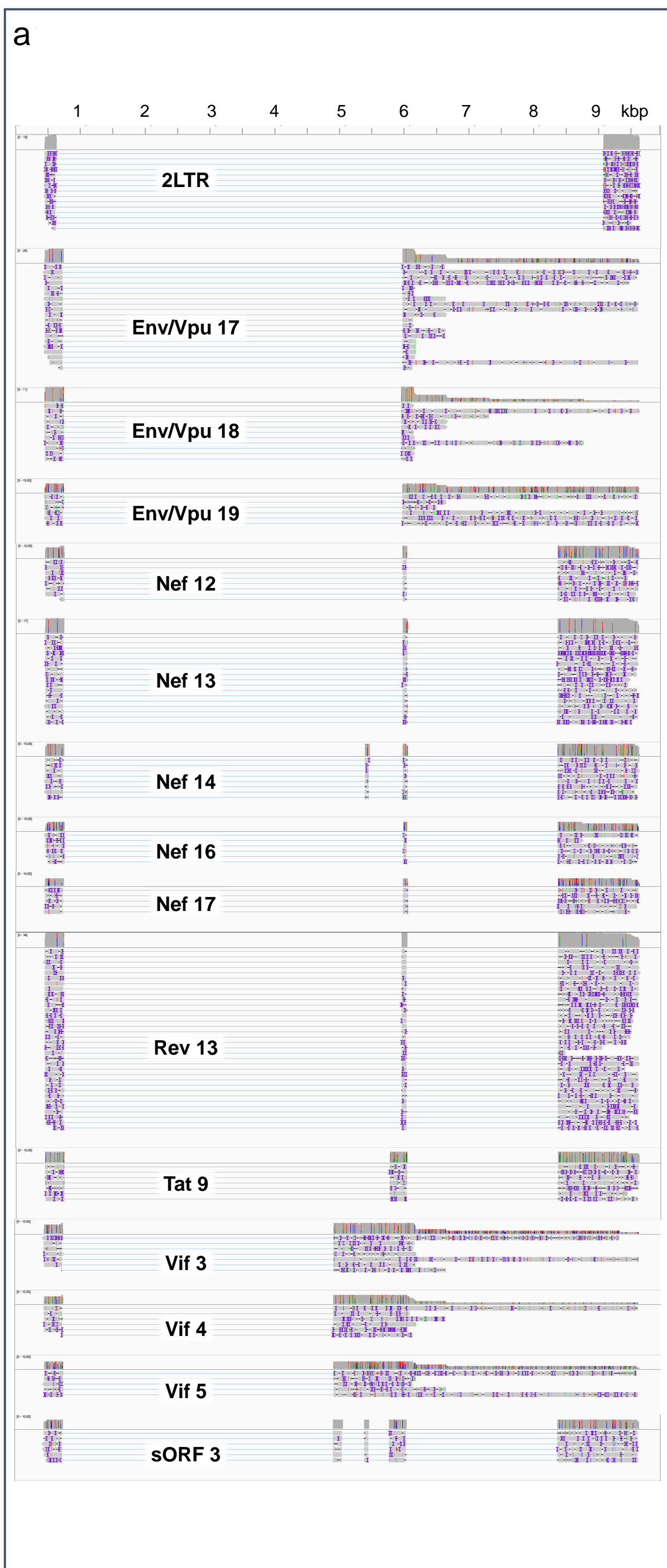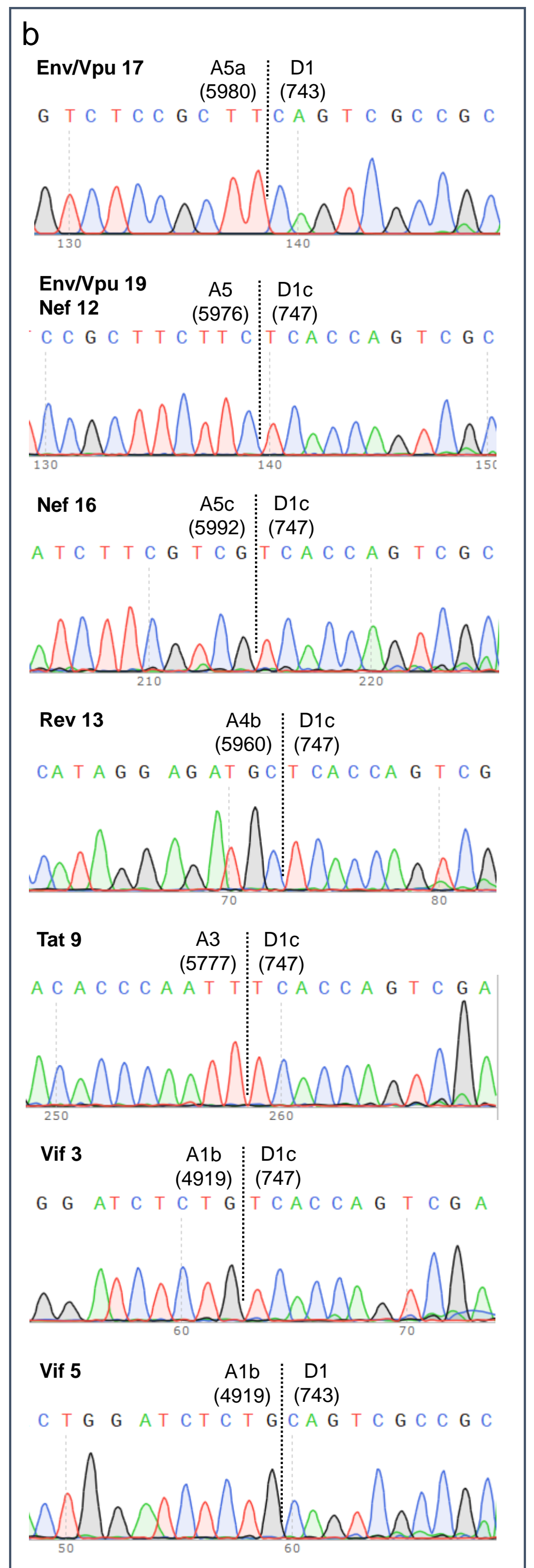

Supplement: Supplementary file 6 — Additional file 6: Figure S2. Identification of new and rare transcripts involving SS D1c, D4a, A1b, A5a and A5c and LTR2 RNA in HIV-1 infected T cell samples. (a) IGV screenshots of ONT read alignments of new and rare transcripts described in the Additional file 4: Table S4. (b) New and rare transcripts were reverse transcribed and amplified using specific primers (Additional file 16: Table S8). DNA sequencing chromatograms of confirmed junctions are presented. [file 12977_2020_533_MOESM6_ESM.pdf]

a

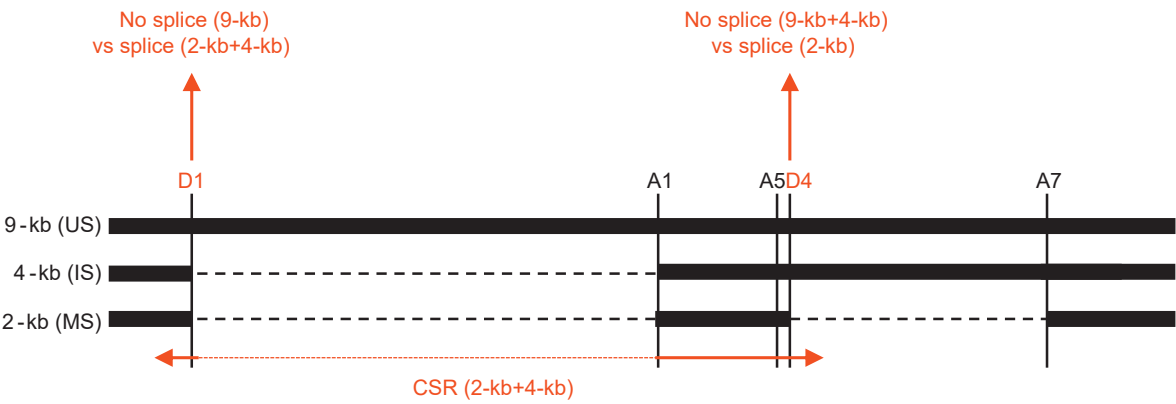

b

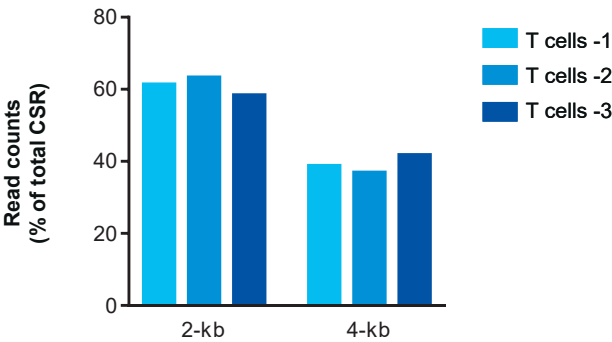

c

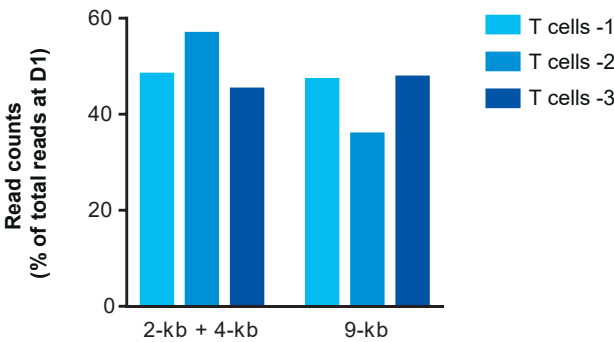

d

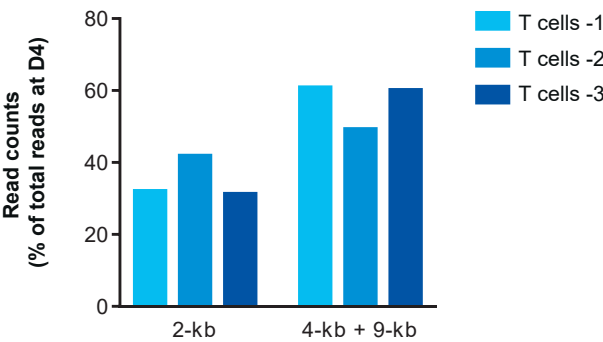

Supplement: Supplementary file 8 — Additional file 8: Figure S3. Estimation of the relative abundance of HIV-1 mRNA size classes using ONT sequencing. (a) Schematic representation of HIV-1 unspliced (US, 9-kb), incompletely spliced (IS, 4-kb) and multiply-spliced (MS, 2-kb) classes of HIV-1 RNA. SS used to calculate the levels of HIV-1 classes are indicated. Excised introns are represented as dotted lines and conserved exons as filled lines. Complete sequencing reads (CSR) corresponding to annotated reads starting before D1, ending after D4 and harbouring a least one splice junction involving D1 are indicated. (b) Relative quantification of MS and IS isoforms were calculated by dividing the number of CSR including (2-kb) or not (4-kb) a splice junction at D4 by the total number of CSR. (c) Relative quantification of 9-kb and spliced RNAs at D1 were estimated by counting the number of reads splicing (2-kb+4-kb) or not (9-kb) at D1 by the total number of reads passing through D1. (d) Relative quantification of 2-kb and 9-kb or 4-kb RNAs were estimated by counting the number of reads splicing (2-kb) or not (4-kb+9-kb) at D4 by the total number of reads passing through D4. Relative level of 9-kb, 4-kb and 2-kb RNAs in Fig. 3 was estimated by integrating the levels of each class determined in (b), (c) and (d). [file 12977_2020_533_MOESM8_ESM.pdf]

## Slide 1
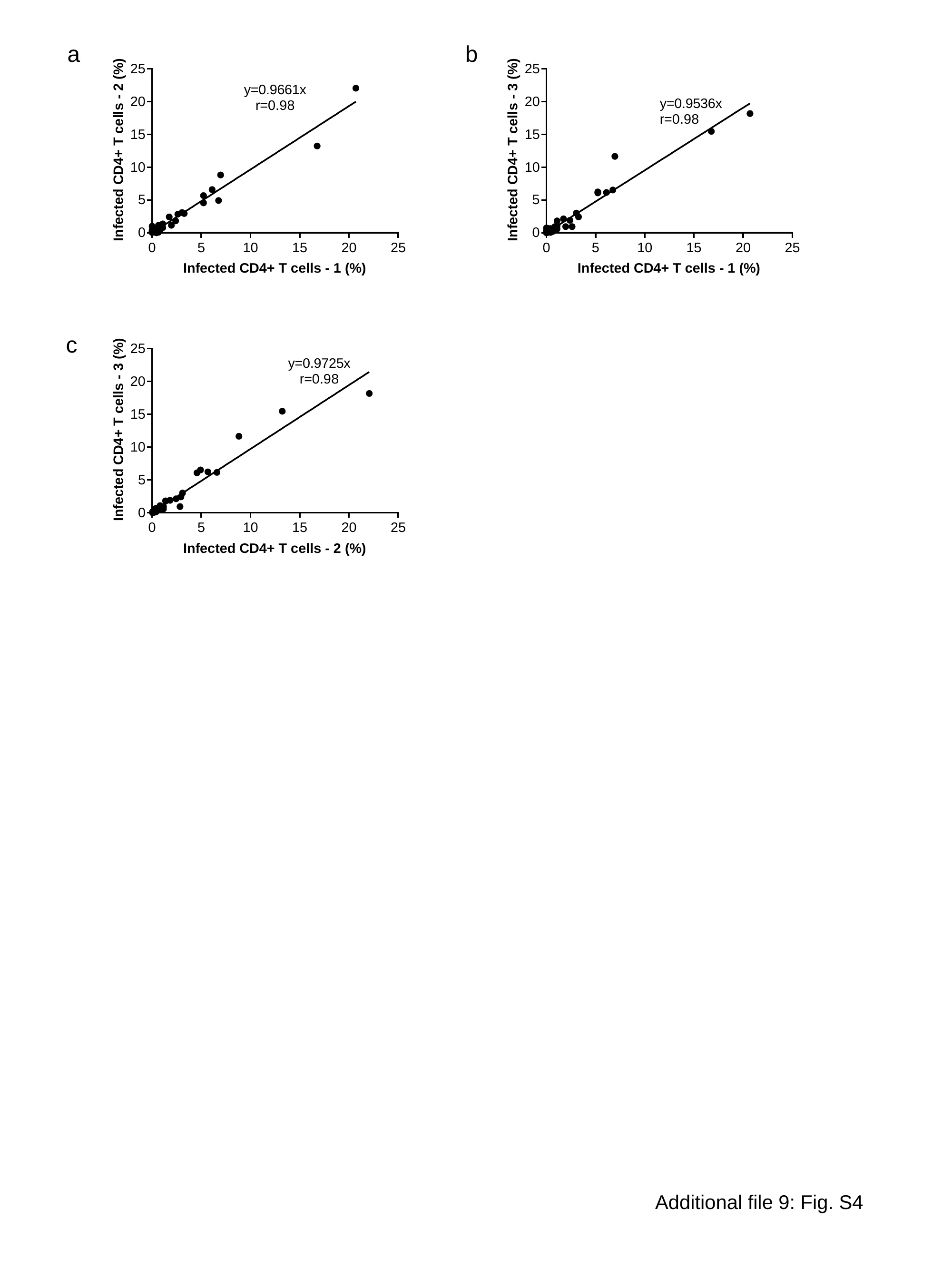

a
b
c
Additional file 9: Fig. S4

Supplement: Supplementary file 9 — Additional file 9: Figure S4. Correlation of viral isoform abundances quantified by ONT sequencing between T cell replicates. The relative abundances of HIV-1 spliced RNAs were calculated as a % of the total number of spliced viral RNAs. Results were compared between infected T cell samples obtained from 3 different donors using a linear regression model supplied by Prism 7: (a) donor 1 vs donor 2; (b) donor 1 vs donor 3; (c) donor 2 vs donor 3. Pearson correlation coefficients r are indicated. p<0.0001. [file 12977_2020_533_MOESM9_ESM.pptx]

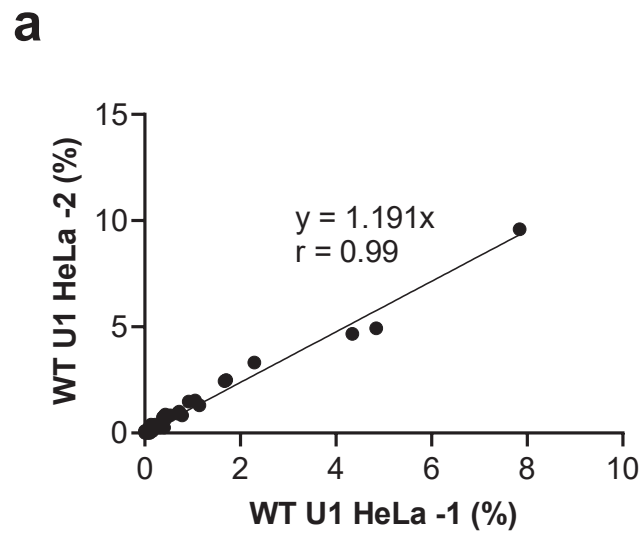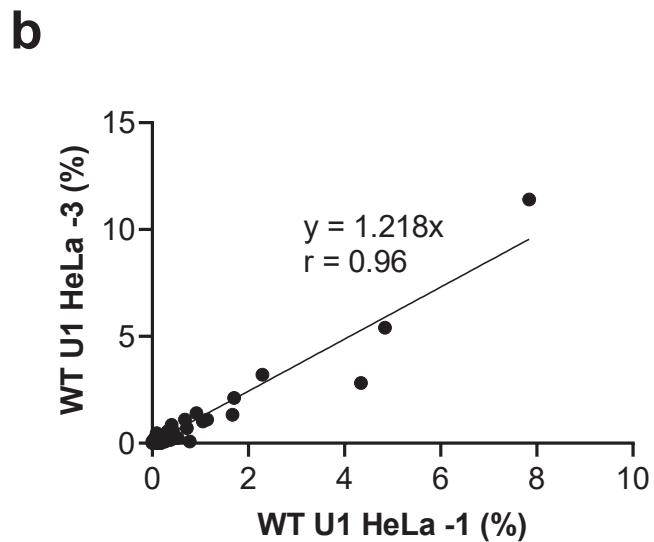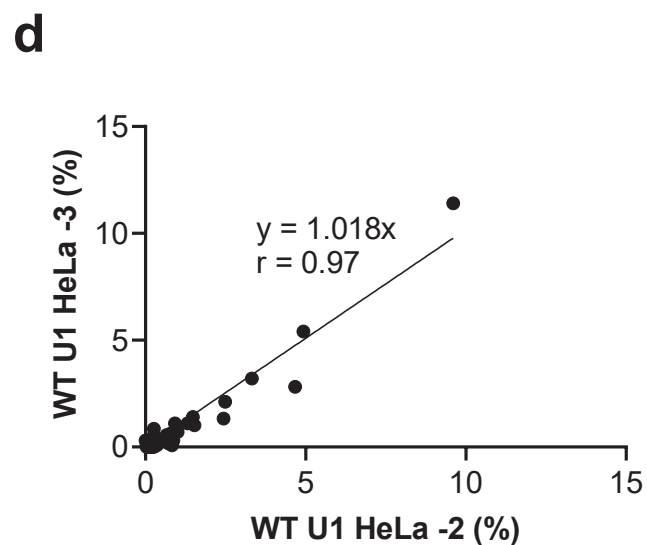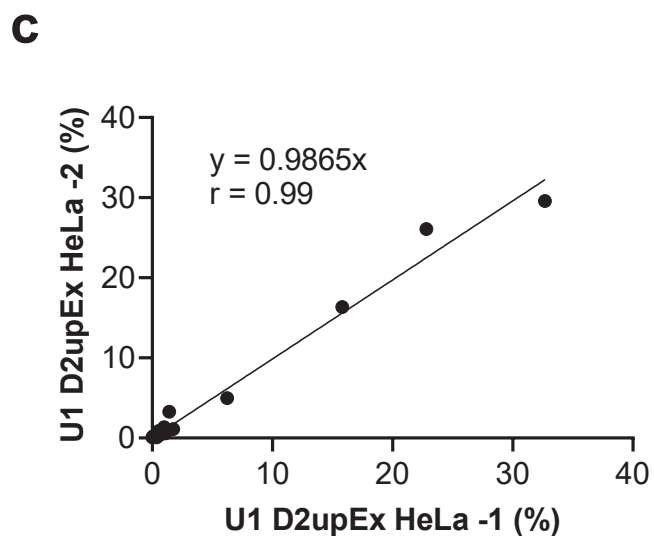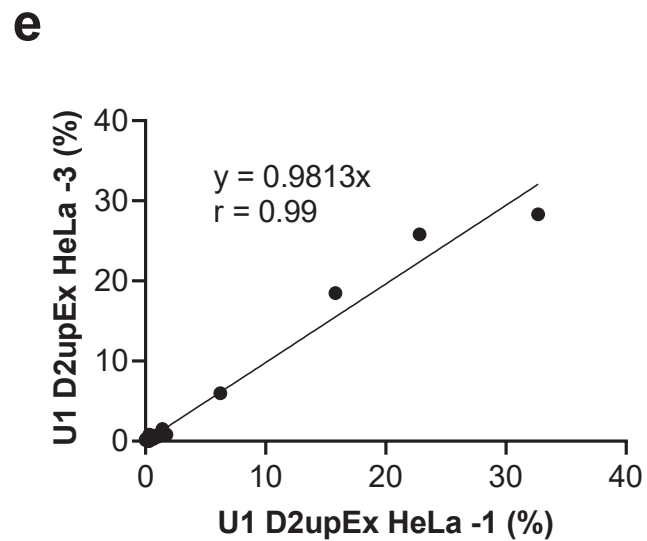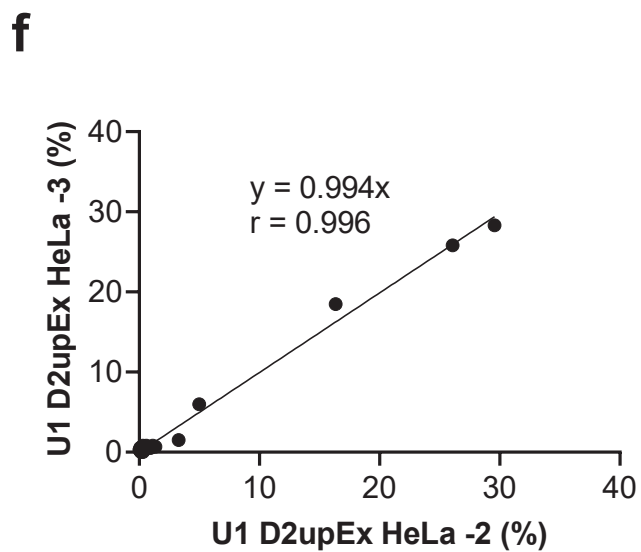

Supplement: Supplementary file 12 — Additional file 12: Figure S7. Correlation of viral isoform abundances quantified by ONT sequencing between biological replicates of HeLa cells expressing either wild-type or U1 D2upEx snRNA. Relative abundances of HIV-1 spliced RNAs were calculated as a % of the total number of viral annotated reads. Results were compared using a linear regression model supplied by Prism 7 between WT U1 HeLa cells samples: (a) sample 1 vs sample 2; (b) sample 1 vs sample 3; (c) sample 2 vs sample 3, and between U1 D2upEx HeLa samples : (d) sample 1 vs sample 2; (e) sample 1 vs sample 3; (f) sample 2 vs sample 3. Pearson correlation coefficients r are indicated. p<0.0001. [file 12977_2020_533_MOESM12_ESM.pdf]

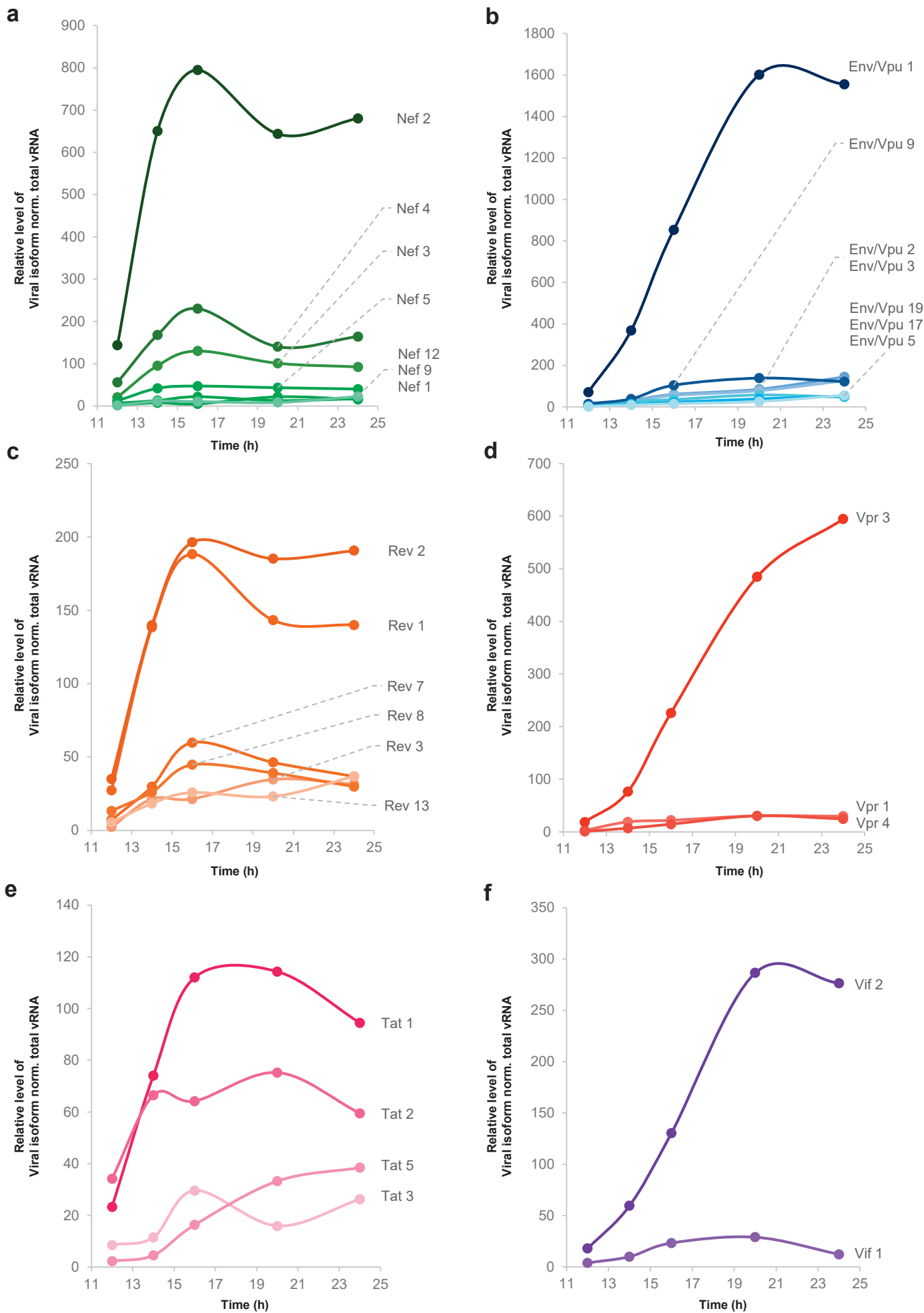

Additional file 15: Fig.S8

Supplement: Supplementary file 15 — Additional file 15: Figure S8. Relative abundance of viral transcripts expressed at early time points of HIV-1 infection in CD4+ T cells, determined by ONT sequencing. Abundance of all viral transcripts expressed in CD4+ T cells from donor 4 between 12 h and 24 hpi was determined as in Fig. 6. Each panel corresponds to a family of transcripts: (a) Nef, (b) Env/Vpu, (c) Vpr, (d) Rev, (e) Tat and (f) Vif. [file 12977_2020_533_MOESM15_ESM.pdf]
