# Supplementary material for: Dynamic nanopore long-read sequencing analysis of HIV-1 splicing events during the early steps of infection
Source: Retrovirology. 2020 Aug 17;17:25. doi: 10.1186/s12977-020-00533-1 (PMC7433067; doi:10.1186/s12977-020-00533-1)
Supplement: Supplementary file 10 — Additional file 10: Figure S5. Relative abundances of viral isoforms in infected and transfected HeLa cells. (a) Relative levels of viral RNA classes in HeLa cell were estimated as described for infected T cells (Fig. 3 and Additional file 8: Fig. S3). (b) Correlation of viral isoform abundances expressed in infected T cells versus transfected HeLa cells according to ONT sequencing. (c) Correlation of viral isoform abundances expressed in infected T cells versus infected HeLa cells according to ONT sequencing. Pearson correlation coefficients r are indicated. p<0.0001. [file 12977_2020_533_MOESM10_ESM.pptx]

## Slide 1
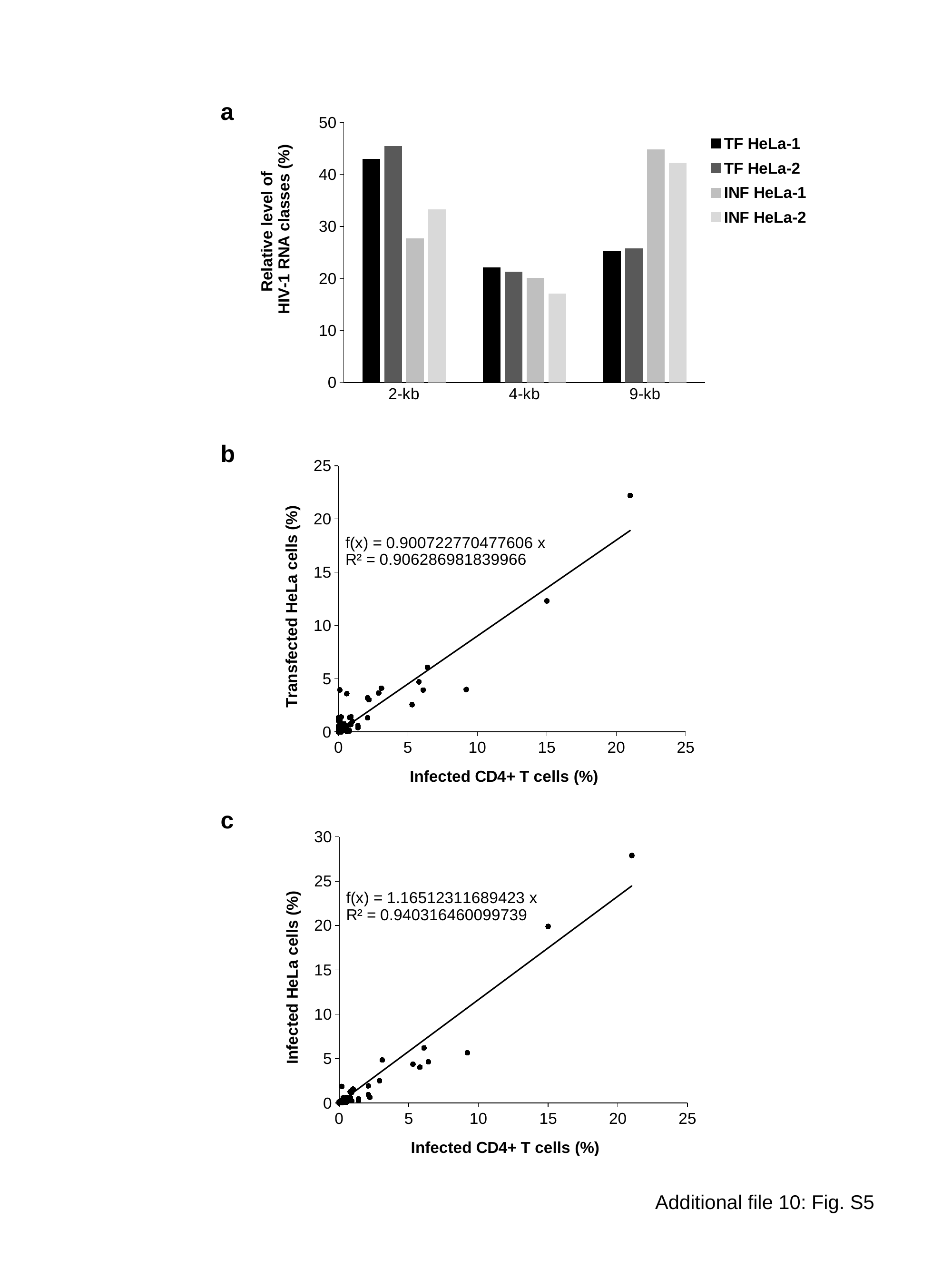

a
### Chart
| Category | TF HeLa-1 | TF HeLa-2 | INF HeLa-1 | INF HeLa-2 |
|---|---|---|---|---|
| 2-kb | 42.953536781744624 | 45.38597817140374 | 27.699399571793233 | 33.24595905172414 |
| 4-kb | 22.04194650642158 | 21.246622748371422 | 20.083475045943768 | 17.02747844827586 |
| 9-kb | 25.158084914182474 | 25.753704649974452 | 44.743883792048926 | 42.1875 |b
### Chart
| Category | |
|---|---|c
### Chart
| Category | INF |
|---|---|Additional file 10: Fig. S5
