# Supplementary material for: Dynamic nanopore long-read sequencing analysis of HIV-1 splicing events during the early steps of infection
Source: Retrovirology. 2020 Aug 17;17:25. doi: 10.1186/s12977-020-00533-1 (PMC7433067; doi:10.1186/s12977-020-00533-1)
Supplement: Supplementary file 11 — Additional file 11: Figure S6. Comparison of relative viral RNA abundances assessed by ONT sequencing and semi-quantitative PCR and gel analysis. Relative abundances of viral isoforms determined by ONT sequencing in Fig. 3 were expressed as a % of the total number of transcripts within either (a) MS RNAs or (b) IS RNAs. Results were compared with quantifications obtained in NL4-3 HIV-1 spreading infection of PBMC by semi-quantitative RT-PCR and gel analysis in [4]. Correlation curves using a linear regression model supplied by Prism 7 are shown and Pearson correlation coefficients r are indicated. p<0.0001. [file 12977_2020_533_MOESM11_ESM.pdf]

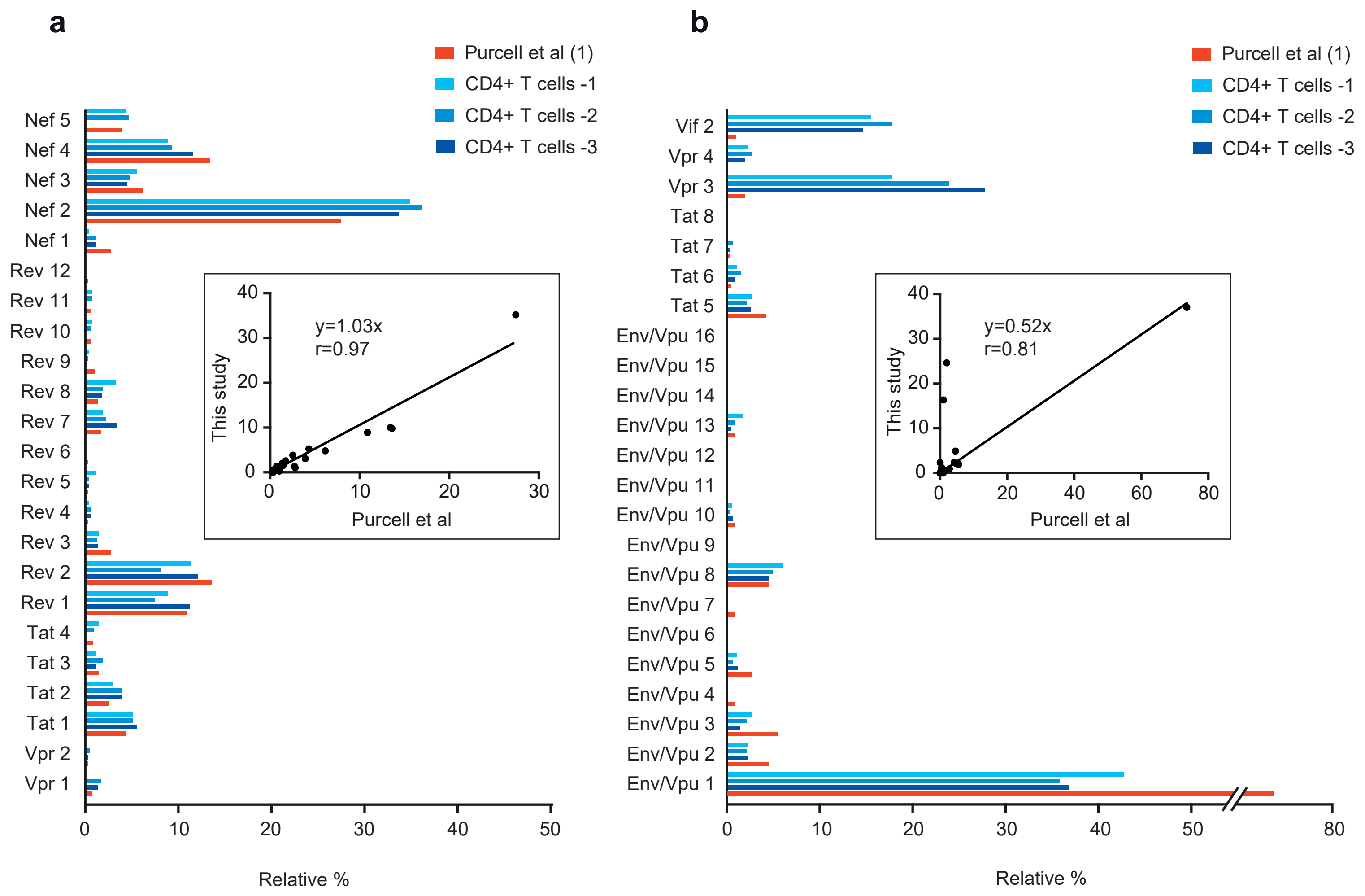

(1). Purcell,D.F.J. and Martin,M.A. (1993) Alternative Splicing of Human Immunodeficiency Virus Type 1 mRNA Modulates Viral Protein Expression, Replication, and Infectivity. J. Virol.

Additional file 11: Fig.S6
